# Superfluid density and collective modes of fermion superfluid in dice lattice

**DOI:** 10.1038/s41598-021-93007-z

**Published:** 2021-06-30

**Authors:** Yu-Rong Wu, Xiao-Fei Zhang, Chao-Fei Liu, Wu-Ming Liu, Yi-Cai Zhang

**Affiliations:** 1 School of Physics and Materials Science, Guangzhou University, Guangzhou, 510006 China; 2 Key Laboratory of Time and Frequency Primary Standards, National Time Service Center, Chinese Academy of Sciences, Xi’an, 710600 China; 3 School of Astronomy and Space Science, University of Chinese Academy of Sciences, Beijing, 100049 China; 4 School of Science, Jiangxi University of Science and Technology, Ganzhou, 341000 China; 5 Beijing National Laboratory for Condensed Matter Physics, Institute of Physics, Chinese Academy of Sciences, Beijing, 100190 China; 6 Songshan Lake Materials Laboratory, Dongguan, Guangdong 523808 China

**Keywords:** Condensed-matter physics, Bose-Einstein condensates

## Abstract

The superfluid properties of attractive Hubbard model in dice lattice are investigated. It is found that three superfluid order parameters increase as the interaction increases. When the filling factor falls into the flat band, due to the infinite large density of states, the resultant superfluid order parameters are proportional to interaction strength, which is in striking contrast with the exponentially small counterparts in usual superfluid (or superconductor). When the interaction is weak, and the filling factor is near the bottom of the lowest band (or the top of highest band), the superfluid density is determined by the effective mass of the lowest (or highest) single-particle band. When the interaction is strong and filling factor is small, the superfluid density is inversely proportional to interaction strength, which is related to effective mass of tightly bound pairs. In the strong interaction limit and finite filling, the asymptotic behaviors of superfluid density can be captured by a parabolic function of filling factor. Furthermore, when the filling is in flat band, the superfluid density shows a logarithmic singularity as the interaction approaches zero. In addition, there exist three undamped collective modes for strong interactions. The lowest excitation is gapless phonon, which is characterized by the total density oscillations. The two others are gapped Leggett modes, which correspond relative density fluctuations between sublattices. The collective modes are also reflected in the two-particle spectral functions by sharp peaks. Furthermore, it is found that the two-particle spectral functions satisfy an exact sum-rule, which is directly related to the filling factor (or density of particle). The sum-rule of the spectral functions may be useful to distinguish between the hole-doped and particle-doped superfluid (superconductor) in experiments.

## Introduction

The superfluid properties in a multi-band (or multi-component) system have been attracting a great interests^1–4^. In comparing with single-band superfluid counterpart, a lot of novel physics, for example, existence of gapped Leggett modes^5–9^, color superconductor^10–12^, coexistence of superfluid and ferromagnetic polarizations^13,14^, induced effective interaction^15^, breaking of Galilean invariance in spin-orbit coupled Bose–Einstein condensation (BEC)^16^, etc, would appear.

For a single flat band system, due to the divergence of effective mass^17^, it is believed that the flat system usually has no superfluidity, e.g., the superfluid density (which is superfluid weight up to a constant) $\rho _s=0$ . However, for a multi-band system, the situation may be quite different^18,19^. In contrast to the single-band system, the superfluid density in a multi-band system can be divided into two part contributions. One of that consists of the diagonal matrix elements of current operator (conventional part), and the other one is the off-diagonal terms (the so called geometric part^20–22^). Even through the conventional part may vanish in flat band limit, the geometric one may be finite. Furthermore, it is found that under some conditions, the geometric part of superfluid density is related to the geometric quantity of energy band, namely, the geometric metric tensor of Bloch states^23^.

**Figure 1:**
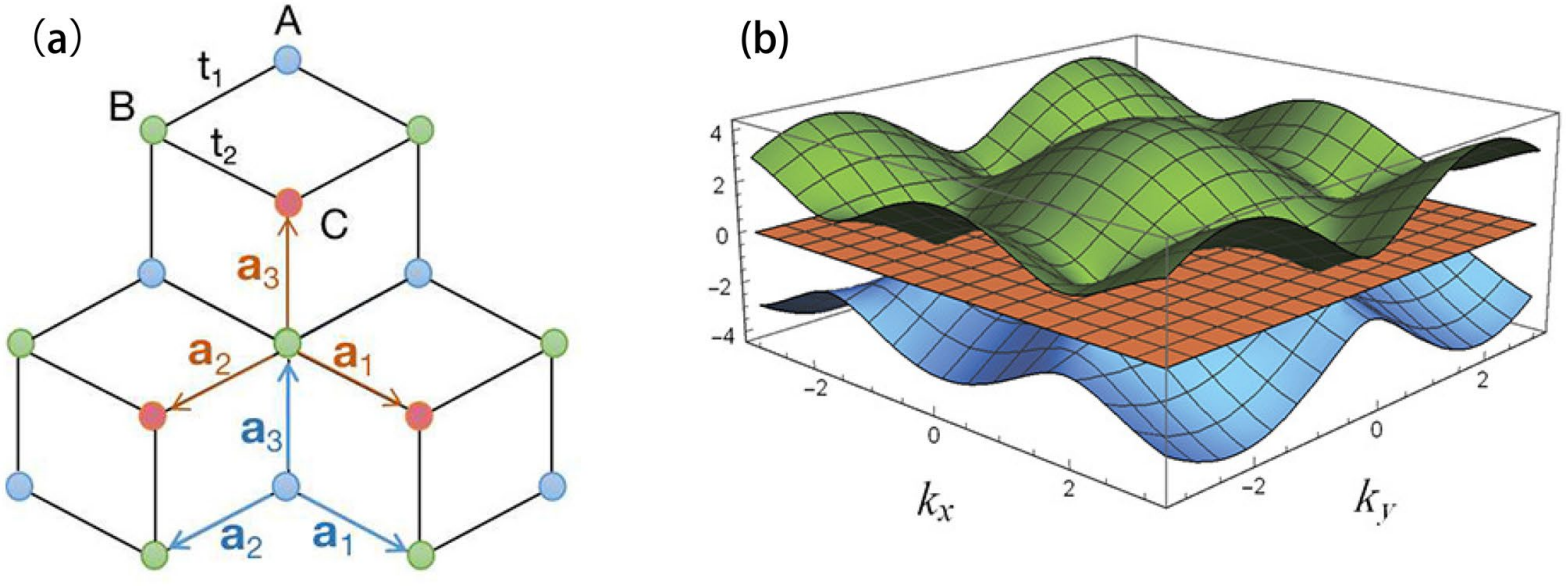
(**a**) The structure of dice lattices. Sublattices A, B and C are shown in blue, green and red respectively. The vectors $\mathbf{a }_1=[\sqrt{3}/2,-1/2]$, $\mathbf{a }_2=[-\sqrt{3}/2,-1/2]$ and $\mathbf{a }_3=[0,1]$ with lattice spacing $a\equiv 1$. (**b**) The three single-particle energy bands with $t=1$ and $\mu =0$ in Eq. (3).

A flat band can appear in dice ($T_3$ ) lattices, similar as graphene, which also has the honeycomb lattice structure (see Fig. 1). In comparing with the graphene, there is an extra lattice site (per unit cell) in the center of hexagon of honeycomb lattice^24–26^. The low-energy physics of the dice lattice are also described by the Dirac equation, but its pseudospin $S=1$ instead of $\frac{1}{2}$^27,28^. There are two schemes to obtain this kind of lattices: one is to fabricate the trilayer structure of cubic lattice (e.g. $SrTiO_3/ SrlrO_3/ SrTiO_3$) in the (111) direction^29^, and the other one is to use the cold atoms in the optical lattices^30^. The band structure of the dice lattices has a distinctive characteristic compared to the graphene, in addition to the conduction band and valence band $\varepsilon _\pm (\mathbf{k })$, there is a flat band $\varepsilon _0 (\mathbf{k })$ in the middle. For the highest (or lowest) band in the Brillouin Zone, there exists two Dirac points, which closely attach to the flat band. Following the discovery of this new material, a lot of novel physical phenomena, for example, Hofstadter butterfly effect^31^, magneto-optical conductivity^32^, Super-Klein tunneling^33^, zitterbewegung effect^34^, magnetism^35^, and magnetoconductivity^36,37^, etc., have attracted great interests.

Although the superfluid density in three band Lieb lattices has been studied^18^, there are still other open questions worthwhile to investigate. For example, what roles does the effective mass play in superfluidity? How do the flat band and Dirac point affect the superfluid order parameters and superfluid density? The collective modes are also interesting subjects in such a three band system. There usually exists several superfluid order parameters in multi-band system. The oscillations between different order parameters result in the so-called Leggett modes^5^. The Leggett modes in condensed system have been observed experimentally in multi-band superconductor $MgB_2$, by using Raman spectroscopy^38^, by angle-resolved photoemission spectroscopy^39^. For three band superfluid in dice lattices, it is expected there may exist several Leggett modes.

In this work, we study the superfluid properties and collective modes of attractive Hubbard model in dice lattices. It is found that the flat band has important influences on the superfluid order parameters and superfluid density. In addition, there exists three collective modes, one of that is the gapless phonon mode, the other two are gapped Leggett modes. The collective modes can be reflected by sharp peaks in two-particle spectral functions. Furthermore, an exact sum-rule of spectral functions is derived, which may be useful to distinguish between the hole-doped and particle-doped superfluid (or superconductor) in experiments.

## Results

The structure of dice lattices is depicted in Fig. 1, every unit cell consists of three different lattice sites (A, B and C), in which sites A and B are located at the vertices of hexagon to form honeycomb lattice, and site C is located at the center of the hexagon. In this work, we only consider the nearest neighbor hopping and equal hopping amplitude, i.e., $t_{1}=t_{2}=t$ (see Fig. 1). The Hubbard model in which two-component Fermi gases with spin $\sigma =\uparrow ,\downarrow$ interacting through on-site attractive interaction $-U$ ($U>0$) is

1 $$\begin{aligned} {\mathscr {H}}=\sum _{\langle ij\rangle \sigma }t_{ij} \psi _{i\sigma }^\dagger \psi _{j\sigma } -\mu \sum _{i\sigma }n_{i\sigma }-U\sum _in_{i\uparrow }n_{i\downarrow }, \end{aligned}$$

where $\mu$ describes the chemical potential. In the following, we would use *i* to label the unit cell. The creation operators for three sub-lattices are represented by $\psi _{iA}^\dagger$ , $\psi _{iB}^\dagger$ and $\psi _{iC}^\dagger$, respectively. $n_{i\sigma }=\psi _{i\sigma }^\dagger \psi _{i\sigma }$ is the particle number operator. Under periodic boundary condition , using a Fourier transform, it is convenient to obtain the non-interacting Hamiltonian

2 $$\begin{aligned} {\mathscr {H}}_0(\mathbf{k})=\sum _{\mathbf{k }} \psi ^\dagger (\mathbf{k}) h(\mathbf{k})\psi (\mathbf{k}), \end{aligned}$$

with $$\psi ^\dagger (\mathbf{k})=[\psi _{A}^\dagger (\mathbf{k}), \psi _{B}^\dagger (\mathbf{k}), \psi _{C}^\dagger (\mathbf{k})]$$ and

3 $$\begin{aligned} h(\mathbf{k})=\left[ \begin{array}{ccc} -\mu &{}h_{12}(\mathbf{k}) &{} 0\\ h_{21}(\mathbf{k}) &{}-\mu &{}h_{23}(\mathbf{k})\\ 0 &{}h_{32}(\mathbf{k}) &{} -\mu \end{array}\right] \end{aligned}$$

where $h_{12}(\mathbf{k})=t\sum _\delta \cos (\mathbf{k}\cdot \mathbf{a}_\delta )+it\sum _\delta \sin (\mathbf{k}\cdot \mathbf{a}_\delta )$, $h_{21}(\mathbf{k})=h^{*}_{12}(\mathbf{k})$, $h_{23}(\mathbf{k})=h_{12}(\mathbf{k})$, $h_{32}(\mathbf{k})=h_{21}(\mathbf{k})$, and $\mathbf{k}=(k_x,k_y)$. The two dimensional vectors $\mathbf{a}_\delta$ are shown in Fig. 1. We note that it is a multi-band (three-band) system and there exist a flat band [$\varepsilon _0(\mathbf{k })=0$] in between the highest [$\varepsilon _+(\mathbf{k })$] and the lowest bands [$\varepsilon _-(\mathbf{k })=-\varepsilon _+(\mathbf{k })$] (see Fig. 1).

It is worth noting that an important role played by particle-hole symmetry in this model. Similarly as Haldane–Hubbard model^40^, applying a particle-hole transformation to the system,

4 $$\begin{aligned} \psi _{i\sigma }\rightarrow \varepsilon _i \psi _{i\sigma }^\dagger , ~~ \psi ^\dagger _{i\sigma }\rightarrow \varepsilon _i \psi _{i\sigma }, \end{aligned}$$

where $\varepsilon _i=1$ for the A and C sublattices, and for the B sublattices $\varepsilon _i=-1$, the hopping term in Hamiltonian Eq. (1) is invariant under the above transformation. After adding some constant terms, the interaction term remains invariant and chemical potential term changes a sign. At this point, we only need to consider the case where the number of particles $n>3$ ($n=3$ is half-filling, i.e. there are three particles in a unit cell). For filling $\Delta n\equiv n-3\ge 0$ (particle-doped case) and $-\Delta n=3-n\le 0$ (hole-doped case), the chemical potentials satisfy a relationship

5 $$\begin{aligned} \mu (-\Delta n)=-\mu (\Delta n). \end{aligned}$$

**Figure 2:**
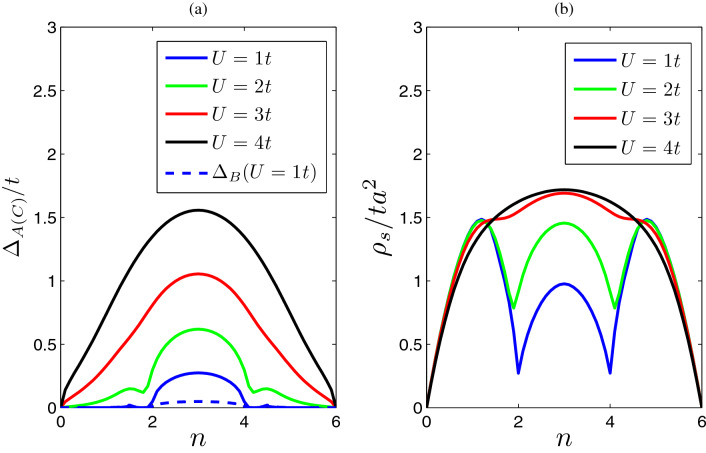
The superfluid order parameters for sublattice A(C) and superfluid densities as functions of filling factor *n*. (**a**) The superfluid order parameters for interaction $U=1$, $U=2$, $U=3$ and $U=4$ are shown in blue, green, red and black solid lines, respectively. The blue dashed line is the order parameter $\Delta _B$ when $U=1$. (**b**) The superfluid densities for interaction $U=1$, $U=2$, $U=3$ and $U=4$ are also shown in blue, green, red and black solid lines, respectively.

It indicates that, at half-filling ($n=3$), the chemical potential is exactly zero. Furthermore, we will see that all the other physical quantities, e.g., superfluid order parameters, superfluid density, etc., are also symmetrical with respect to the half-filling $n=3$ (see Fig. 2).

The order parameters ($\Delta _i=-U\langle \psi _{i\downarrow }\psi _{i\uparrow }\rangle$) can be calculated with mean field theory (see “Methods”). The evolutions of the superfluid order parameters are depicted in panel (a) of Fig. 2. It is found that $\Delta _i$ is always real. The order parameters are symmetrical with respect to the half-filling due to the particle-hole symmetry. The Hamiltonian is subject to the inversion symmetry, $\Delta _A$ is the same as $\Delta _C$ but differ from $\Delta B$. As one increases the interaction *U*, $\Delta _ i$ always increases. The order parameters can increase without limit as long as the interaction is strong enough. For example, when filling factor is half-filling ($n=3$), $\Delta _i\simeq U/2$ as $U\rightarrow \infty$, which is a half of the energy of two particle bound states^41^.

When the filling factor approaches zero, the order parameters decrease to zero. The fully occupied case ($n=6$) is equivalent to completely empty ($n=0$) case due to the particle-hole symmetry. With the increasing of filling factor *n*, the $\Delta _i$ gets larger. However, when the filling factor is in between the lowest band and the middle flat band, the order parameter gets a dip due to the small density of states near the Dirac points [especially for weakly interacting cases in panel (a) of Fig. 2]. The suppressions of order parameters are also found in Lieb lattices^42^. When the filling factor lies in the middle region (e.g., $n\simeq 3$), the order parameter reaches its maximum due to enhancement of density of states in flat band.

The interaction effects amplified by band flatness can give rise to Wigner-crystal state^43^ and ferromagnetic transition^44^. Similarly, the behaviors of superfluid order parameters are also affected significantly by the flat band^45^. For example, when the filling factor is in the middle of the flat band (e.g., half filling $n=3$), the infinite large density of states results in order parameter $\Delta _A=\Delta _C\simeq U/4$ as the interaction approaches zero, which is very different from that in the usual Bardeen–Cooper–Schrieffer (BCS) superconductor (or superfluid), where pairing gap $\Delta$ is exponentially small when interaction $U\rightarrow 0$. In addition, for half-filling $n=3$, it is found that the order parameter for sublattice B is much smaller than $\Delta _{A(C)}$ when the interaction is small ($U/t\ll 1$), i.e., $\Delta _B/\Delta _{A(C)}\ll 1$ (see Fig. 2). This is because the weights of sublattices A and C in flat band wave function is dominant over that of sublattice B.

### Superfluid density

The superfluid density can be calculated with phase twist method^20,46^. Assuming the superfluid order parameters undergo a phase variation, e.g, $\triangle _i\rightarrow \Delta _ie^{2i\mathbf{q }\cdot \mathbf{r }_i}$, the superfluid density (particle number per unit cell) tensor $\rho _{sij}$ can be written as

6 $$\begin{aligned} \rho _{sij}= \frac{\partial ^2 \Omega (\mathbf{q })}{\partial q_i \partial q_i}|_{q\rightarrow 0}, \end{aligned}$$

where $\Omega$ is thermodynamical potential (per unit cell) in grand canonical ensemble. Similarly as the Haldane model^20^, due to the $C_3$ symmetry of honeycomb lattices^19,47^, the superfluid density tensor can be simplified into a scalar, i.e., $\rho _{sij}=diag\{\rho _s,\rho _s\}$.

The superfluid density $\rho _s$ is plotted as a function of filling factor for different interactions in panel (b) of Fig. 2. First of all, we see that the superfluid density is also symmetrical with respect to the half-filling due to the particle-hole symmetry. When the filling factor is completely empty ($n\rightarrow 0$) [or fully occupied case ($n\rightarrow 6$)], the superfluid density vanishes linearly. For the small filling factor ($n\ll 1$) and weak interaction ($U\ll t$) case (BCS-limit^48^), the superfluid density can be written as

7 $$\begin{aligned} \rho _s(U/t\ll 1)=n/m^*=3tn/(\sqrt{2})\simeq 2.12nt, \end{aligned}$$

where $m^*=\sqrt{2}/(3t)$ is the effective mass near the bottom of lowest band, which is determined by the single-particle energy band structure, and does not depend on interaction strength *U*. The superfluid density is proportional to filling factor (or particle density) and inversely proportional to the effective mass of energy band^17^, which is very similar to that of Bose–Einstein condensate with spin-orbital coupling^49^.

Differently from order parameters, when interaction $U/t\gg 1$, the superfluid density remains finite. In such case, the superfluid consists of tightly bound pairs. Two bound pairs could not occupy a same lattice site due to the constraint of exclusive principle of fermions. So the pairs can be viewed as hard-core bosons, which is BEC limit of fermion superfluid. When the number of the effective hard-core bosons is small $n\ll 1$ (dilute gas limit), the superfluid density is determined by the effective mass of tightly bound pairs, i.e.,

8 $$\begin{aligned} \rho _s(U/t\gg 1)=n'/m'^*=8t^2n/U, \end{aligned}$$

where $n'=n/2$ is filling factor of effective hard core bosons and $m'^*=U/(16t^2)$ is its effective mass, which is determined by the dispersion of two-particle bound state energy (Note $m'^*\ne 2m^*$ due to the coupling of between the motions of center of mass and relative motion of two particles in lattice^50^). The above equation indicates that when the interaction gets stronger (in strong interaction limit), the effective mass becomes larger, the resultant superfluid density gets smaller. For a moderate interaction strength (e.g., $U/t>5$), and small filling ($n\ll 1$), it is expected that the superfluid density should satisfy $8t^2n/U<\rho _s<2.12nt$.

**Figure 3:**
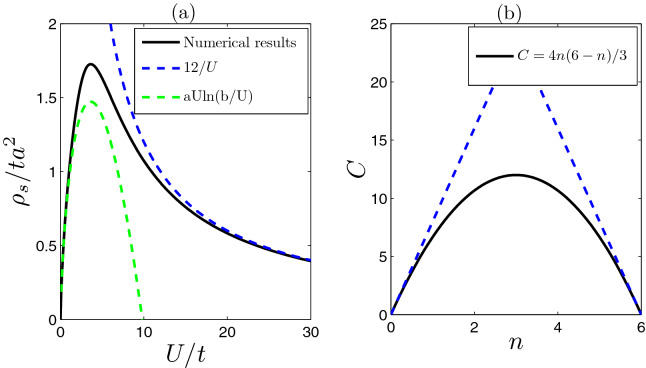
The asymptotic behaviors of superfluid densities. (**a**) The numerical results of superfluid density are plotted in black solid line (filling factor $n=3$). The two dashed lines correspond two asymptotic limits of superfluid density, which are given by $\rho _s\simeq 12/U$ for $U\rightarrow \infty$) and *aUln*(*b*/*U*) with $a=0.4077$ and $b=9.822$ for $U\rightarrow 0$, respectively. (**b**) The blue dashed line is the estimated up bound 8*n* (filling factor $0\le n\le 3$), which is determined by effective mass of tightly bound pairs [$m^{'*}=U/(16t^2)$]. Using the particle-hole symmetry, the corresponding up bound for $3\le n\le 6$ also depicted here. The solid line is the parabolic function Eq. (11).

In addition, differently from the order parameters, the evolution trends of superfluid density with the interactions can bedifferent for different regimes of filling factors. For example, when the filling is small, superfluid density diminishes with the increasing of interaction, which is opposite to that of order parameters [see panel (b) of Fig. 2]. Only when the filling is sufficiently large (for example, $n\simeq 3$), the superfluid density grows up as the interaction increases (within a certain range of interaction, see the following discussions and the panel (a) of Fig. 3). Due to the vanishingly small density of states near the Dirac points, the superfluid density also develops a dip for weak interacting cases. Similarly as Lieb lattices^18^, a triple dome structure appears in the superfluid density, especially for weakly interacting cases [see panel (b) of Fig. 2].

For intermediate filling factor (e.g, $n=3$ in flat band), it is found that the superfluid density approaches zero as $U\rightarrow 0$. Once the interaction is turned on, the superfluid density grows rapidly [see panel (a) of Fig. 3]. Specially here, the non-vanishing superfluid density for weak interactions mainly arises from the contributions of off-diagonal matrix elements of current operator (the geometric part). In the isolated flat band limit^18,22^ or under the assumption of uniform pairing^1,20^, the superfluid density in flat band can be related to geometric metric tensor of Bloch sates. However, here we note that the superfluid density approaches zero according to the law of

9 $$\begin{aligned} \rho _s=aUln[b/U], \end{aligned}$$

as $U\rightarrow 0$ with two constants $a\simeq 0.4077$ and $b=9.822$ for half filling $n=3$ [see panel (a) in Fig.3], which is different from the linear dependence in isolated flat band limit^18^. As $U\rightarrow 0$, the appearance of the logarithmic singularity (*UlnU*) is attributed to the existences of the gapless Dirac points and vanishingly small pairing gap ($\Delta \propto U\rightarrow 0$). This is because when filling factor is in the middle of flat band (half-filling of $n=3$) and $U\rightarrow 0$ (or $\Delta \equiv \Delta _A=\Delta _C\simeq U/4 \rightarrow 0$), it is found that the superfluid density mainly arises from matrix elements of current operator between the flat band and other bands near the Dirac points (the off-diagonal part). Furthermore, the contribution near the Dirac points can be approximated by

10 $$\begin{aligned} \rho _s\sim A\Delta \int _{0}^{B} \frac{ qdq}{\sqrt{q^2+\Delta ^2}\left( \sqrt{q^2+\Delta ^2}+3\Delta \right) }=A\Delta ln\frac{\sqrt{B^2+\Delta ^2}+3\Delta }{4\Delta }\simeq a U ln(b/U), \end{aligned}$$

where *A*, *B*, *a* and *b* are four constants. A similar logarithmic singularity of superfluid density also appear in the Mielke checkerboard lattices^51^.

With the increasing of interaction, the superfluid density reaches its maximum values at $U\simeq 3.7t$ [see panel (a) in Fig. 3]. After passing over that point, it decreases. For strong interaction limit and finite *n*, the trend of evolutions of superfluid density can be also understood qualitatively by effective mass of tightly bound pairs. Considering the fermion nature of tightly bound pairs, it is expected that the superfluid density should be smaller than that determined by $m'^*$, namely, $\rho _s< 8t^2n/U$ for BEC limit ($U\gg t$). For example, when $n=3$, it is found that the superfluid density is also inversely proportional to interaction, i.e., $\rho _s\rightarrow C/U$ as $U\rightarrow \infty$ [see panel (a) of Fig. 3]. The numerical results give the constant $C\simeq 12$ for half-filling case ($n=3$), which is almost a half of the estimated up bound [$8t^2n=24$ (t=1)]. Nevertheless, the up bound would provide much more precise estimation for superfluid density when filling factor *n* is small [see panel (b) of Fig. 3]. For a general filling *n*, it is found that the constant *C* can be given by a parabolic function (see “Methods”), namely

11 $$\begin{aligned} C=\frac{4t^2}{3}n(6-n). \end{aligned}$$

Consequently, the superfluid density

12 $$\begin{aligned} \rho _s=\frac{4t^2}{3U}n(6-n). \end{aligned}$$

The asymptotic results Eqs. (11) and (12) in strong interaction limit are very similar to the behaviors of the superfluid density of hard-core bosons in lattices^52,53^.

### Collective modes

In this section, we would present Gaussian fluctuation method^54–56^ to calculate the collective modes and spectral functions^40^. The order parameters can be decomposed as mean-field part and fluctuation, i.e., $\Delta _i=\Delta _i+\delta \Delta _i$ with $i=1,2,\ldots m$, and *m* is the number of order parameters. The partition function can be written as

13 $$\begin{aligned}&Z\approx e^{-S_0}\int D \eta ^{\dag }_q D\eta _q e^{-\delta S}, \end{aligned}$$

where $S_0$ is the mean-field contribution and the Gaussian fluctuation part (see “Methods”)

14 $$\begin{aligned}&\delta S=\frac{1}{2}\sum _{\mathbf{q },n}\eta ^{\dag }_q M(q)\eta _q=\sum _{\mathbf{q },n>0}\eta ^{\dag }_q M(q)\eta _q, \end{aligned}$$

with pairing fluctuation fields $\eta ^{\dag }_q=[\Delta ^{*}_{1q},\Delta ^{*}_{2q},\ldots ,\Delta ^{*}_{mq},\Delta _{1,-q},\Delta _{2,-q},\Delta _{m,-q}]$ and $q=(\mathbf{q },i\omega _n)$, $\omega _{n}=2n\pi /\beta$ ($n\in Z$) is Matsubara frequency, $\beta =1/T$ is inverse temperature. The fluctuation matrix *M* is a $2m\times 2m$ matrix,

15 $$  \begin{aligned}&M_{ij}(\mathbf{q },i\omega _n)=\frac{1}{\beta }\sum _{\mathbf{k },n'}G^{0}_{ij}(k+q)G^{0}_{j+m,i+m}(k)+\frac{\delta _{ij}}{U}, \qquad \qquad \qquad \qquad (1\le i,j\le m)\nonumber \\&M_{ij}(\mathbf{q },i\omega _n)=\frac{1}{\beta }\sum _{\mathbf{k },n'}G^{0}_{ij}(k+q)G^{0}_{j-m,i+m}(k), \qquad \qquad \qquad \qquad (1\le i\le m \; \& \; m+1\le j\le 2m)\nonumber \\&M_{ij}(\mathbf{q },i\omega _n)=\frac{1}{\beta }\sum _{\mathbf{k },n'}G^{0}_{ij}(k+q)G^{0}_{j+m,i-m}(k), \qquad \qquad \qquad \qquad (m+1\le i\le 2m \;  \&  \; 1\le j\le m)\nonumber \\&M_{ij}(\mathbf{q },i\omega _n)=\frac{1}{\beta }\sum _{\mathbf{k },n'}G^{0}_{ij}(k+q)G^{0}_{j-m,i-m}(k)+\frac{\delta _{ij}}{U}, \qquad \qquad \qquad \qquad (m+1\le i,j\le 2m ), \end{aligned}$$

where

16 $$\begin{aligned} G^{0}_{ij}(k)=([i\omega _{n'}-H_{\mathrm{BdG}}(\mathbf{k })]^{-1})_{ij}, \end{aligned}$$

is matrix element of Nambu–Gorkov Green function. Here $k=(\mathbf{k },i\omega _{n'})$ and $\omega _{n'}=(2n'+1)\pi /\beta$ [$n'\in Z$].

The collective modes are given by zeros of determinant $Det|M(\mathbf{q },i\omega _n\rightarrow \omega +i0^+)|=0$. As $q\rightarrow 0$, the gapless collective mode is the Anderson–Bogoliubov phonon, which characterizes the density oscillations of superfluid. The gapped ones are Leggett modes, which correspond relative oscillations of densities of different sublattices^5–8,40^.

The two-particle normal (anomalous) Green function matrix elements are defined as^47^

17 $$\begin{aligned}&G_{II,ij}(\mathbf{q },i\omega _n)=\sum _n\left[ \frac{\langle 0|\Delta _{i \mathbf{q }}|n\rangle \langle n|\Delta ^{\dag }_{j \mathbf{q }}|0\rangle }{i\omega _n-\omega _{n0}}-\frac{\langle 0|\Delta ^{\dag }_{j \mathbf{q }}|n\rangle \langle n|\Delta _{i \mathbf{q }}|0\rangle }{i\omega _n+\omega _{n0}}\right] ,\nonumber \\&F_{II,ij}(\mathbf{q },i\omega _n)=\sum _n\left[ \frac{\langle 0|\Delta _{j,\mathbf{q }}|n\rangle \langle n|\Delta _{i,-\mathbf{q }}|0\rangle }{i\omega _n-\omega _{n0}}-\frac{\langle 0|\Delta _{i,-\mathbf{q }}|n\rangle \langle n|\Delta _{j,\mathbf{q }}|0\rangle }{i\omega _n+\omega _{n0}}\right] , \end{aligned}$$

where $\omega _{n0}=E_n-E_0$, $E_n$ and $|n\rangle$ are eigenvalues and eigenstates of Hamiltonian, respectively. $\Delta _{i\mathbf{q }}$ is pairing fluctuation operator of superfluid order parameters [see Eq. (22) in the next section]. With the action $\delta S$ (Gaussian weight), the correlation functions (and the matrix elements of two-particle Green functions) can be calculated^47^, i.e.,

18 $$\begin{aligned}&-G_{II,ij}(q)=\langle \Delta ^{*}_{jq} \Delta _{iq}\rangle =(M^{-1})_{i,j},\nonumber \\&-F_{II,ij}(q)=\langle \Delta _{j,-q} \Delta _{iq} \rangle =(M^{-1})_{i,m+j},\nonumber \\&-F^{*}_{II,ji}(q)=\langle \Delta ^{*}_{jq}\Delta ^{*}_{i,-q} \rangle =(M^{-1})_{m+i,m+j},\nonumber \\&-G_{II,ji}(-q)=\langle \Delta _{j,-q}\Delta ^{*}_{i,-q}\rangle =(M^{-1})_{m+i,m+j}. \end{aligned}$$

The two-particle spectral functions can be expressed in terms of the Green functions as^57^

19 $$\begin{aligned}&A_{ij}(\mathbf{q },\omega )=-\frac{1}{\pi }Im [G_{II,ij}(\mathbf{q },i\omega _n\rightarrow \omega +i0^+)],\nonumber \\&B_{ij}(\mathbf{q },\omega )=-\frac{1}{\pi }Im [F_{II,ij}(\mathbf{q },i\omega _n\rightarrow \omega +i0^+)], \end{aligned}$$

where $0^+$ denotes a positive infinitesimal number. In addition, in the presence of inversion symmetry, the superfluid density can be also related to the pairing fluctuation matrix *M*^47^

20 $$\begin{aligned} \rho _s\!\!=\!\!\mathrm{lim_{q\rightarrow 0}}\frac{4}{q^2}(\Delta ^{t*},\Delta ^t).\!\!\left( \!\!\! \begin{array}{cccc} I &{} 0 \\ 0&{} -I\\ \end{array}\!\!\!\right) \!\!.M(q).\!\!\left( \!\!\! \begin{array}{cccc} I &{} 0 \\ 0&{} -I\\ \end{array}\!\!\!\right) \!\!.\!\!\left( \!\!\! \begin{array}{cccc} \Delta \\ \Delta ^*\\ \end{array}\!\!\!\right) . \end{aligned}$$

where $(I)_{m\times m}$ is a $m\times m$ identity matrix, $\Delta =(\Delta _1,\Delta _2,\ldots ,\Delta _m )^t$, and $(\ldots )^t$ denotes matrix transpose. In dice lattice case, the number of order parameters $m=3$ and we identify $\Delta _1=\Delta _A$, $\Delta _2=\Delta _B$ and $\Delta _3=\Delta _C$.

**Figure 4:**
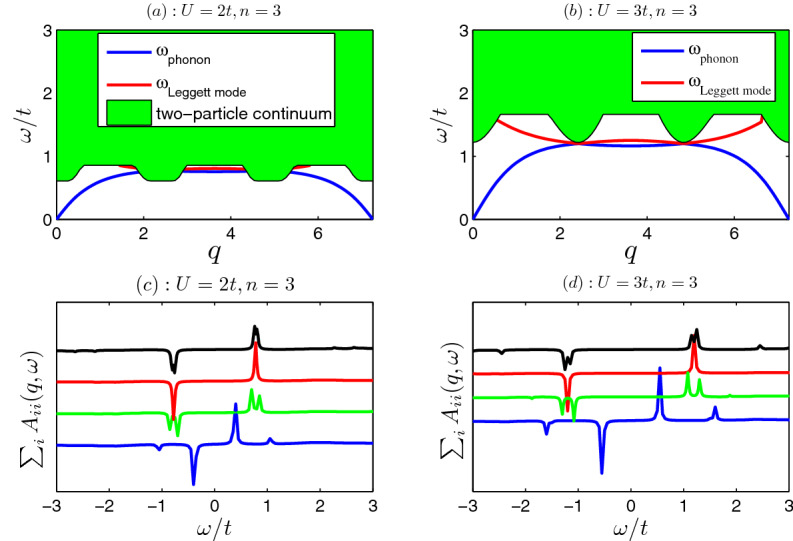
Collective modes (**a**,**b**) in x-direction $\mathbf{q }=q {\hat{x}}$ and two-particle spectral functions (**c**,**d**). (**a**) The phonon (Leggett) mode is plotted in blue (red) line. The green shaded region is the two-particle continuous spectra. The interaction $U=2$, filling factor $n=3$ in (**a**). (**b**) The phonon mode and Leggett mode with $U=3;n=3$. (**c**) Spectral functions with $U=2;n=3$. (**d**) Spectral functions with $U=3;n=3$. The spectral functions of $q=0.5, 1.5, 2.5$ and 3.5 are plotted in blue, green, red and black lines, respectively. The sharp peaks in spectral functions correspond the collective modes. In order to get the spectral functions numerically, we take $0^+=0.02$ in Eq. (19).

**Figure 5:**
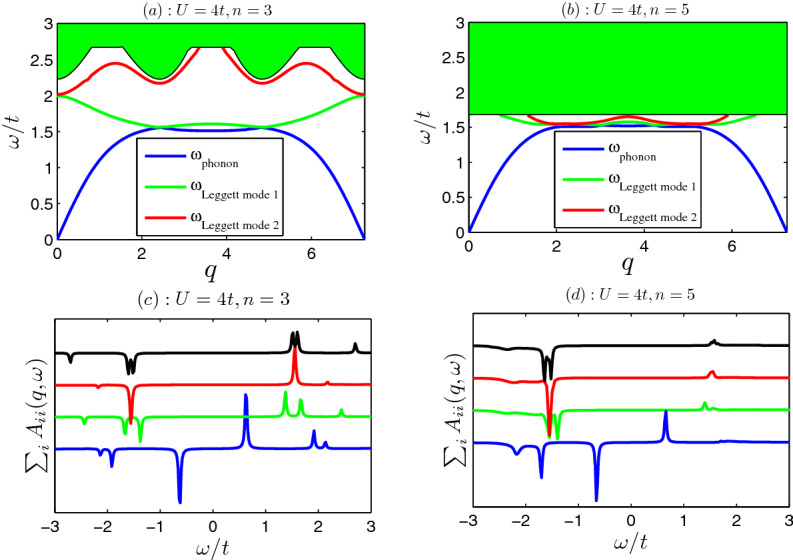
Collective modes (**a**,**b**) in x-direction $\mathbf{q }=q {\hat{x}}$ and two-particle spectral functions (**c**,**d**). (**a**) The phonon (Leggett) mode is plotted in blue (green and red) lines. The interaction $U=4$, filling factor $n=3$ in (**a**). (**b**) The phonon mode and Leggett modes with $U=4;n=5$. (**c**) Spectral functions with $U=4;n=3$. (**d**) Spectral functions with $U=4;n=5$. The spectral functions of $q=0.5, 1.5, 2.5$ and 3.5 are plotted in blue, green, red and black lines, respectively.

Figures 4 and 5 show the collective modes with the increasing of wave vector *q*. It is found that there exist three undamped collective modes for strong interactions [see panels (a) and (b) of Fig. 5]. The lowest one is the gapless phonon as $q\rightarrow 0$, which corresponds to total density oscillation. The upper two gapped excitations are the Leggett modes, which corresponds to the relative density oscillations between sublattices. As the interaction strength decreases, the bottom of two-particle continuum lowers, the region of the existence of collective modes shrinks. The collective modes merge into the continuum and becomes damped [see panels (a) and (b) of Fig. 4]. As a result of that, the collective modes are not well defined. Only when the interaction is strong enough, the undamped Leggett modes survive (see Fig. 5). The collective modes also reflected in the two-particle spectral functions as shown in Figs. 4 and 5. The sharp peaks in spectral functions correspond the collective modes [see panels (c) and (d) in Figs. 4, 5]. The phonon is in-phase oscillation of three order parameters, while the other two Leggett modes are out-phase ones.

### Two-particle spectral functions

The two-particle spectral functions of a general superfluid state can be also defined as

21 $$\begin{aligned}&A_{\alpha \beta ,\gamma \delta }(\mathbf{q },\omega )=\sum _n\langle 0|\Delta _{\alpha \beta \mathbf{q }}|n\rangle \langle n|\Delta ^{\dag }_{\gamma \delta \mathbf{q }}|0\rangle \delta (\omega -\omega _{n0})-\sum _n\langle 0|\Delta ^{\dag }_{\gamma \delta \mathbf{q }}|n\rangle \langle n|\Delta _{\alpha \beta \mathbf{q }}|0\rangle \delta (\omega +\omega _{n0}),\nonumber \\&B_{\alpha \beta ,\gamma \delta }(\mathbf{q },\omega )=\sum _n\langle 0|\Delta _{\alpha \beta \mathbf{q }}|n\rangle \langle n|\Delta _{\gamma \delta , -\mathbf{q }}|0\rangle \delta (\omega -\omega _{n0})-\sum _n\langle 0|\Delta _{\gamma \delta , -\mathbf{q }}|n\rangle \langle n|\Delta _{\alpha \beta \mathbf{q }}|0\rangle \delta (\omega +\omega _{n0}), \end{aligned}$$

where $\omega _{n0}=E_n-E_0$, $E_n$ and $|n\rangle$ are eigenvalues and eigenstates of Hamiltonian, respectively. $\Delta _{\alpha \beta \mathbf{q }}$ is pairing fluctuation operator of superfluid order parameters, i.e.,

22 $$\begin{aligned} \Delta _{\alpha \beta \mathbf{q }}=-U\sum _\mathbf{k } \psi _{\alpha \mathbf{k }+\mathbf{q }}\psi _{\beta -\mathbf{k }}, \end{aligned}$$

where $\psi _{\alpha \mathbf{k }+\mathbf{q }}$ fermion field operator for single particle state $\alpha$ in momentum space, which satisfies anti-commutation relation

23 $$\begin{aligned} \psi _{\alpha \mathbf{k }}\psi ^{\dag }_{\beta \mathbf{k }'}+\psi ^{\dag }_{\beta \mathbf{k }'}\psi _{\alpha \mathbf{k }}=\delta _{\alpha \beta }\delta _{\mathbf{k }\mathbf{k }'}. \end{aligned}$$

The two-particle normal (anomalous) Green functions can be expressed in terms of spectral functions, i.e.,

24 $$\begin{aligned}&G_{II\alpha \beta ,\gamma \delta }(\mathbf{q },i\omega _n)=\int _{-\infty }^{+\infty } d\omega \frac{A_{\alpha \beta ,\gamma \delta }(\mathbf{q },\omega )}{i\omega _n-\omega },\nonumber \\&F_{II\alpha \beta ,\gamma \delta }(\mathbf{q },i\omega _n)=\int _{-\infty }^{+\infty } d\omega \frac{B_{\alpha \beta ,\gamma \delta }(\mathbf{q },\omega )}{i\omega _n-\omega }. \end{aligned}$$

Similarly as single-particle spectral function^58^, the two-particle spectral functions satisfies a sum-rule, i.e.,

25 $$\begin{aligned}&\int d\omega A_{\alpha \beta ,\gamma \delta }(\mathbf{q },\omega )=\left\langle 0|[\Delta _{\alpha \beta \mathbf{q }},\Delta ^{\dag }_{\gamma \delta \mathbf{q }}] |0\right\rangle =U^2\left\langle 0|\sum _\mathbf{k }[\delta _{\beta \delta }\psi _{\alpha \mathbf{k }}\psi ^{\dag }_{\gamma \mathbf{k }}-\delta _{\alpha \gamma }\psi ^{\dag }_{\delta \mathbf{k }}\psi _{ \beta \mathbf{k }}]|0\right\rangle \nonumber \\&\quad =U^2\left\langle 0|\sum _\mathbf{k }[\delta _{\beta \delta }\delta _{\alpha \gamma }-\delta _{\beta \delta }\psi ^{\dag }_{\gamma \mathbf{k }}\psi _{\alpha \mathbf{k }}-\delta _{\alpha \gamma }\psi ^{\dag }_{\delta \mathbf{k }}\psi _{ \beta \mathbf{k }}]|0\right\rangle ,\nonumber \\&\int d\omega B_{\alpha \beta ,\gamma \delta }(\mathbf{q },\omega )= \langle 0|[\Delta _{\alpha \beta \mathbf{q }},\Delta _{\gamma \delta -\mathbf{q }}] |0\rangle =0. \end{aligned}$$

Taking the diagonal elements ($\gamma =\alpha ;\delta =\beta$),

26 $$\begin{aligned}&\int d\omega A_{\alpha \beta ,\alpha \beta }(\mathbf{q },\omega )=\left\langle 0|[\Delta _{\alpha \beta \mathbf{q }},\Delta ^{\dag }_{\alpha \beta \mathbf{q }}] |0\right\rangle =U^2\left\langle 0|\sum _\mathbf{k }[\psi _{\alpha \mathbf{k }}\psi ^{\dag }_{\alpha \mathbf{k }}-\psi ^{\dag }_{\beta \mathbf{k }}\psi _{ \beta \mathbf{k }}]|0\right\rangle \nonumber \\&\quad =U^2\left\langle 0|\sum _\mathbf{k }[1-\psi ^{\dag }_{\alpha \mathbf{k }}\psi _{\alpha \mathbf{k }}-\psi ^{\dag }_{\beta \mathbf{k }}\psi _{ \beta \mathbf{k }}]|0\right\rangle =U^2[N_{cell}-(N_\alpha +N_\beta )], \end{aligned}$$

where $N_{cell}$ is the number of unit cells, $N_\alpha$ is the particle number of $\alpha$-th component.

In our dice lattice case, there exist three order parameters and they can be identified as $\Delta _{A(B /C)\downarrow A(B /C)\uparrow }=\Delta _{A(B / C)}$. The sum-rule is

27 $$\begin{aligned}&\int d\omega B_{i,j}(\mathbf{q },\omega )= \langle 0|[\Delta _{i \mathbf{q }},\Delta _{j -\mathbf{q }}] |0\rangle =0,\nonumber \\&\int d\omega A_{i,j}(\mathbf{q },\omega )=\langle 0|[\Delta _{i \mathbf{q }},\Delta ^{\dag }_{j \mathbf{q }}] |0\rangle =U^2[N_{cell}-(N_{i\downarrow }+N_{i\uparrow })]\delta _{ij}=U^2[N_{cell}-N_i]\delta _{ij}, \end{aligned}$$

where indices $i,j=A,B,C$, particle number of *i*-th sublattice $N_{i}=N_{i\uparrow }+N_{i\downarrow }$.

If we take the diagonal elements of $A_{ij}$ and sum over *i*, then we get a much more useful sum-rule

28 $$\begin{aligned}&A\equiv \int d\omega \sum _{i}A_{i,i}(\mathbf{q },\omega )=\sum _{i}\langle 0|[\Delta _{i \mathbf{q }},\Delta ^{\dag }_{i \mathbf{q }}] |0\rangle =U^2\sum _{i}[N_{cell}-N_i]=U^2[3N_{cell}-N]\propto -(n-3), \end{aligned}$$

where total particle number $N=N_A+N_B+N_c$ and filling factor $n=N/N_{cell}$. It should be emphasized that the above sum-rule of spectral functions is exact, which does not depend on what state the system is. One can use the above sum-rule to check the calculations of various theories.

The above Eq. (28) shows that the integral of spectral functions is proportional to filling factor $n-3$ (relative to the half filling). If the filling factor is half filling ($n-3=0$), the integral of spectral functions is exactly zero. While when filling factor is larger (smaller) than half-filling [particle (hole)-doped case], the integral of spectral functions is negative (positive). Our numerical calculations verify the above observations. For example, when the filling is half-filling ($n=3$), we see that the spectral functions are odd functions of frequency and the integral of spectral functions vanishes exactly [see panels (c, d) of Fig. 4 and panel (c) of Fig. 5]. Panel (d) of Fig. 5 shows the spectral functions for particle-doped case ($n=5$), the weight of negative frequency $\omega <0$ is dominant, and the integral of spectral functions is negative. The above findings may be useful to distinguish between the hole-doped and particle-doped superfluid or superconductor in experiments.

In addition, for a fixed $\mathbf{q }$, when $\omega$ approaches some non-degenerate collective modes (e.g., phonon or Leggett modes), from the definition Eq. (21), the spectral functions should take the following forms

29 $$\begin{aligned}&A_{i,i}(\mathbf{q },\omega )=\langle 0|\Delta _{i \mathbf{q }}|n\rangle \langle n|\Delta ^{\dag }_{i\mathbf{q }}|0\rangle \delta (\omega -\omega _{n0}),\nonumber \\&A_{i,j}(\mathbf{q },\omega )=\langle 0|\Delta _{i \mathbf{q }}|n\rangle \langle n|\Delta ^{\dag }_{j\mathbf{q }}|0\rangle \delta (\omega -\omega _{n0}),\nonumber \\&A_{j,j}(\mathbf{q },\omega )=\langle 0|\Delta _{j \mathbf{q }}|n\rangle \langle n|\Delta ^{\dag }_{j\mathbf{q }}|0\rangle \delta (\omega -\omega _{n0}), \end{aligned}$$

where $|n\rangle =|\mathbf{q }\rangle$ is wave function of the collective mode, $\omega _{n0}$ is its excitation energy. As a result of that, when $\omega \simeq \omega _{n0}$, the heights of peaks of two-particle spectral functions near the collective mode should satisfy a relation, i.e.,

30 $$\begin{aligned} |A_{i,j}(\mathbf{q },\omega )|^2= A_{i,i}(\mathbf{q },\omega )A_{j,j}(\mathbf{q },\omega ). \end{aligned}$$

When *q* is small ($q\rightarrow 0$), the matrix elements of two-particle Green functions of zero frequency $\omega =0$ are proportional to product of two order parameters, namely^47^

31 $$\begin{aligned}&G_{II,ij}(\mathbf{q },0)\simeq -4Z\Delta _{i}\Delta ^*_{j},\nonumber \\&F_{II,ij}(\mathbf{q },0)\simeq 4Z\Delta _{i}\Delta _{j}, \end{aligned}$$

where $Z\equiv \sum _n[\frac{|\langle 0|{\hat{\theta }}_{\mathbf{q }}|n\rangle |^2}{\omega _{n0}}+\frac{|\langle 0|{\hat{\theta }}_{-\mathbf{q }}|n\rangle |^2}{\omega _{n0}}]\simeq 1/(q^2\rho _s)>0$ is a real number and ${\hat{\theta }}_{\mathbf{q }}$ the phase operator^59,60^. In such a case, it is expected that, for small *q*, the two-particle spectral functions near the phonon states [$\omega \simeq \omega _{phonon}(\mathbf{q })$ or $\omega \simeq -\omega _{phonon}(-\mathbf{q })$] are also proportional to product of superfluid order parameters, i.e.,

32 $$\begin{aligned}&A_{ij}(\mathbf{q },\omega )\simeq 4Z_+ \Delta _{i}\Delta ^*_{j}\delta [\omega -\omega _{phonon}(\mathbf{q })]-4Z_- \Delta _{i}\Delta ^*_{j}\delta [\omega +\omega _{phonon}(-\mathbf{q })],\nonumber \\&B_{i,j}(\mathbf{q },\omega )\simeq -4Z_+ \Delta _{i}\Delta _{j}\delta [\omega -\omega _{phonon}(\mathbf{q })]+4Z_- \Delta _{i}\Delta _{j}\delta [\omega +\omega _{phonon}(-\mathbf{q })], \end{aligned}$$

where $Z_+=|\langle 0|{\hat{\theta }}_{\mathbf{q }}|\mathbf{q }\rangle |^2$ and $Z_-=|\langle 0|{\hat{\theta }}_{-\mathbf{q }}|-\mathbf{q }\rangle |^2$ are two real number, which are approximately equal as $q\rightarrow 0$.

**Table 1:** The verifications of Eqs. (30) and (32) of spectral functions near the phonon modes with $U=4$ and filling factor $n=5$ [same as that in panel (b) and (d) of Fig. 5].

1	2	3	4	5	6	7	8	9
q	$\omega$	$A_{11}$	$A_{12}$	$A_{22}$	$\frac{A_{11}}{\Delta _{1}^{2}}$	$\frac{A_{12}}{\Delta _{1}\Delta _2}$	$\frac{A_{22}}{\Delta _{2}^{2}}$	$\frac{A_{12}^2}{A_{11}A_{22}}$
0.01	0.018	1274.5	1618.3	2054.8	2306.5	2318.3	2330.0	1.00002
0.1	0.15	129.4	167.9	217.8	234.2	240.5	247.0	1.0002
0.5	0.66	24.17	34.01	47.82	43.75	48.72	54.22	1.0007

Our numerical calculations indeed verify Eqs. (30) and (32) for the case of $q\rightarrow 0$ . For example, some numerical results of spectral functions are listed in Table 1. It is found that, for extremely small wave vector ($q=0.01$), both Eqs. (30) and (32) are satisfied approximately. As *q* increases from $q=0.01$ to $q=0.1$ and eventually $q=0.5$, Eq. (32) becomes invalid gradually (see the columns 6, 7, 8 in the Table 1). Nevertheless, Eq. (30) still holds (see the last column of the Table 1).

## Discussion

In conclusion, we investigate the superfluid properties of attractive Hubbard model in dice lattice. The order parameters and superfluid density as functions of filling factors and interactions are analyzed in great detail. We emphasize the important roles of effective masses in the understanding of the asymptotic behaviors of superfluid density, especially for small filling factor. When both the filling factor and interaction are small, the superfluid density is given by the effective mass of single particle energy band. When interaction is strong, the superfluid density is inversely proportional to interaction, which is also related to effective mass of tightly bound pairs. At strong interaction limit, the asymptotic behavior of superfluid density is captured by a parabola function of filling factor. In addition, the flat band has significant influences on superfluidity. To be specific, the infinite large density of states of flat band results in a linear interaction-dependence of superfluid order parameters. When interaction is weak, the existences of Dirac points and vanishingly small order parameters cause a logarithmic singularity of superfluid density.

Due to the existence of three order parameters, there are three collective modes, i.e., one is the gapless phonon, which corresponds to total density oscillations; the others are gapped Leggett modes, which are characterized by the relative density oscillations between different sublattices. It is found that the undamped Leggett modes can exist in strong interaction cases. In addition, the collective modes can be reflected by sharp peaks in the two-particle spectral functions. The behaviors of two-particle spectral functions near the phonon modes are also analyzed in detail. In addition, an exact sum-rule of spectral functions is derived. For theoretical aspects, the sum-rule can be used to check the various theoretical calculations. In experiments, the sum-rule of the spectral functions may be useful to distinguish between the hole-doped and particle-doped superfluid (or superconductor).

## Methods

### Mean field theory

The Hamiltonian Eq. (1) can be solved with the mean-field theory. The superfluid order parameter (pairing gap) $\Delta _{A(B,C)}=-U\langle \psi _{iA(B/C)\downarrow }\psi _{iA(B/C)\uparrow }\rangle$. The Bogoliubov–de Gennes Hamiltonian:

33 $$\begin{aligned} {\mathscr {H}}_{\mathrm{BdG}}=\sum _\mathbf{k}\Psi ^\dagger (\mathbf{k})H_{\mathrm{BdG}}(\mathbf{k})\Psi (\mathbf{k}), \end{aligned}$$

which $$\Psi ^\dagger (\mathbf{k})\!\!=\!\![\psi _{A\uparrow }^\dagger (\mathbf{k}),\psi _{B\uparrow }^\dagger (\mathbf{k}),\psi _{C\uparrow }^\dagger (\mathbf{k}),\psi _{A\downarrow }(-\mathbf{k}),\psi _{B\downarrow }(-\mathbf{k}),\psi _{C\downarrow }(-\mathbf{k})]$$ and

34 $$\begin{aligned} H_{\mathrm{BdG}}(\mathbf{k })\!\!=\!\!\left[ \!\!\begin{array}{cccccc} {\begin{matrix} -\mu &{} h_{12}(\mathbf{k}) &{} 0 &{}\Delta _{\mathrm{A}} &{} 0 &{} 0\\ h_{21}(\mathbf{k}) &{} -\mu &{}h_{23}(\mathbf{k}) &{} 0 &{} \Delta _{\mathrm{B}} &{} 0\\ 0 &{} h_{32}(\mathbf{k}) &{} -\mu &{} 0 &{} 0 &{} \Delta _{\mathrm{C}}\\ \Delta ^*_{\mathrm{A}} &{} 0 &{} 0 &{}+\mu &{} -h_{12}^*(-\mathbf{k}) &{} 0\\ 0 &{} \Delta ^*_{\mathrm{B}} &{} 0 &{} -h_{21}^*(-\mathbf{k}) &{} \mu &{}-h^*_{23}(-\mathbf{k})\\ 0 &{} 0 &{} \Delta ^*_{\mathrm{C}} &{} 0 &{} -h_{32}^*(-\mathbf{k}) &{} +\mu \end{matrix}} \end{array}\!\!\right] . \end{aligned}$$

The Bogoliubov transformation of above equation is readily carried out to obtain the eigenenergies $E^{1,2,3+(-)}(\mathbf{k})$, where $E^{n-}(\mathbf{k })=-E^{n+}(-\mathbf{k })<0$ and 1, 2, 3 represent three branches of energy. The thermodynamic potential per unit cell in the ground state is

35 $$\begin{aligned} \begin{aligned} \Omega =\frac{1}{N_{cell}}\sum _\mathbf{k}[E^{1-}(\mathbf{k})+E^{2-}(\mathbf{k})+E^{3-}(\mathbf{k})-3\mu ]+\frac{(\Delta _{\mathrm{A}}^2+\Delta _{\mathrm{B}}^2+\Delta _{\mathrm{C}}^2)}{U}, \end{aligned} \end{aligned}$$

where $N_{cell}$ is the number of unit cells. The order parameters (or pairing gaps) and chemical potential can be obtained by solving $\partial \Omega /\partial \Delta _{\mathrm{A,B,C}}=0$, and $n=-\partial \Omega /\partial \mu$ consistently. In the whole paper, we set $t=1$ as the energy unit and lattice spacing $a=1$.

### Asymptotic behaviors of superfluid density

For dice lattices, the superfluid order parameters for three sublattices are

36 $$\begin{aligned}&\Delta _{A}=-U\langle \psi _{iA\downarrow }\psi _{iA\uparrow }\rangle ,\nonumber \\&\Delta _{B}=-U\langle \psi _{iB\downarrow }\psi _{iB\uparrow }\rangle ,\nonumber \\&\Delta _{C}= -U\langle \psi _{iC\downarrow }\psi _{iC\uparrow }\rangle , \end{aligned}$$

Using the mean-field decoupling, the BdG Hamiltonian is

37 $$\begin{aligned} {\mathscr {H}}_{\mathrm{BdG}}=\sum _\mathbf{k}\Psi ^\dagger (\mathbf{k})H_{\mathrm{BdG}}(\mathbf{k})\Psi (\mathbf{k}), \end{aligned}$$

where $$\Psi ^\dagger (\mathbf{k})\!\!=\!\![\psi _{A\uparrow }^\dagger (\mathbf{k}),\psi _{B\uparrow }^\dagger (\mathbf{k}),\psi _{C\uparrow }^\dagger (\mathbf{k}),\psi _{A\downarrow }(-\mathbf{k}),\psi _{B\downarrow }(-\mathbf{k}),\psi _{C\downarrow }(-\mathbf{k})]$$ and

38 $$\begin{aligned} H_{\mathrm{BdG}}(\mathbf{k})=\left( \!\!\! \begin{array}{cccccc} h^{p}_{11}(\mathbf{k })&{} h^{p}_{12}(\mathbf{k }) &{} 0&{} \Delta _A &{}0 &{} 0 \\ h^{p}_{21}(\mathbf{k }) &{} h^{p}_{22}(\mathbf{k }) &{}h^{p}_{23}(\mathbf{k }) &{} 0&{}\Delta _B &{} 0 \\ 0 &{} h^{p}_{23}(\mathbf{k }) &{}h^{p}_{33}(\mathbf{k }) &{} 0 &{} 0 &{}\Delta _C\\ \Delta ^{*}_{A} &{}0 &{} 0 &{}-h^{h}_{11}(-\mathbf{k }) &{} -h^{h}_{12}(-\mathbf{k })&{}0 \\ 0 &{}\Delta ^{*}_{B} &{} 0 &{}-h^{h}_{21}(-\mathbf{k }) &{} -h^{h}_{22}(-\mathbf{k })&{}-h^{h}_{23}(-\mathbf{k }) \\ 0 &{}0 &{} \Delta ^{*}_{C} &{}0 &{} -h^{h}_{32}(-\mathbf{k })&{}-h^{h}_{33}(-\mathbf{k }) \\ \end{array}\!\!\!\right) , \end{aligned}$$

where the particle and hole part Hamiltonian are

39 $$\begin{aligned} h^{p}(\mathbf{k })=\left( \!\!\! \begin{array}{ccc} h^{p}_{11}(\mathbf{k })&{} h^{p}_{12}(\mathbf{k }) &{} 0 \\ h^{p}_{21}(\mathbf{k }) &{} h^{p}_{22}(\mathbf{k }) &{}h^{p}_{23}(\mathbf{k })\\ 0 &{} h^{p}_{23}(\mathbf{k }) &{}h^{p}_{33}(\mathbf{k }) \\ \end{array}\!\!\!\right) , \end{aligned}$$

and

40 $$\begin{aligned} h^{h}(-\mathbf{k })=\left( \!\!\! \begin{array}{cccc} h^{h}_{11}(-\mathbf{k }) &{} h^{h}_{12}(-\mathbf{k })&{}0 \\ h^{h}_{21}(-\mathbf{k }) &{} h^{h}_{22}(-\mathbf{k })&{}h^{h}_{23}(-\mathbf{k }) \\ 0 &{} h^{h}_{32}(-\mathbf{k })&{}h^{h}_{33}(-\mathbf{k })\\ \end{array}\!\!\!\right) , \end{aligned}$$

respectively. The above BdG matrix can be diagonalized, e.g.,

41 $$\begin{aligned}&H_{\mathrm{BdG}}(\mathbf{k})|n+,k\rangle =E^{n+}(\mathbf{k })|n+,\mathbf{k }\rangle , \nonumber \\&H_{\mathrm{BdG}}(\mathbf{k})|n-,k\rangle =E^{n-}(\mathbf{k })|n-,\mathbf{k }\rangle , \end{aligned}$$

where quasi-particle energy $E^{n+}(\mathbf{k })=E^{n}(\mathbf{k }))>0$ and $E^{n-}(\mathbf{k })=-E^{n+}(-\mathbf{k })<0$ due to particle-hole symmetry of the Bogoliubov–de Gennes Hamiltonian. For three band dice lattices, the indices $n=1,2,3$. Eigen-states $|n+,\mathbf{k }\rangle$ and $|n-,\mathbf{k }\rangle$ (“quasi-particle basis”) can be also written as

42 $$\begin{aligned}&|n+,\mathbf{k }\rangle =\sum _{s=1,2,3}W^{+}_{ns}(\mathbf{k })|ps,\mathbf{k }\rangle +\sum _{t=1,2,3}W^{+}_{nt}(\mathbf{k })|ht,\mathbf{k }\rangle ,\nonumber \\&|n-,\mathbf{k }\rangle =\sum _{s=1,2,3}W^{-}_{ns}(\mathbf{k })|ps,\mathbf{k }\rangle +\sum _{t=1,2,3}W^{-}_{nt}(\mathbf{k })|ht,\mathbf{k }\rangle , \end{aligned}$$

where $|ps,\mathbf{k }\rangle$ and $|ht,\mathbf{k }\rangle$ are eigen-states of particle and hole part Hamiltonian, respectively, i.e,

43 $$\begin{aligned}&h^{p}(\mathbf{k })|ps,\mathbf{k }\rangle =\varepsilon ^{p}_s(\mathbf{k })|ps,\mathbf{k }\rangle ,\nonumber \\&h^{h}(-\mathbf{k })|ht,\mathbf{k }\rangle =\varepsilon ^{h}_t(\mathbf{k })|ht,\mathbf{k }\rangle . \end{aligned}$$

They are also the “energy band basis” for single-particle Hamiltonian. Then $W^{+}$ and $W^{-}$ are transformation matrices between the above two sets of basis. Due to the time reversal symmetry in dice lattices, $h^{p}(\mathbf{k })=h^{h}(-\mathbf{k })= h(\mathbf {k})$ [ Eq. (3)], $\varepsilon ^{p}_n(\mathbf{k })=\varepsilon ^{h}_n(\mathbf{k })\equiv \varepsilon _n(\mathbf{k })$ and $|pn,\mathbf{k }\rangle =|hn,\mathbf{k }\rangle \equiv |n,\mathbf{k }\rangle$.

The thermodynamic potential per unit cell is

44 $$\begin{aligned} \begin{aligned} \Omega =\frac{1}{N_{cell}}\sum _\mathbf{k}[E^{1-}(\mathbf{k })+E^{2-}(\mathbf{k })+E^{3-}(\mathbf{k })]-3\mu +\frac{(\Delta _{\mathrm{A}}^2+\Delta _{\mathrm{B}}^2+\Delta _{\mathrm{C}}^2)}{U}=\frac{1}{N_{cell}}\sum _{\mathbf{k },n=1,2,3}E^{n-}(\mathbf{k })+Cons, \end{aligned} \end{aligned}$$

where $N_{cell}$ is the number of unit cells. The constant $Cons=-3\mu +\frac{(\Delta _{\mathrm{A}}^2+\Delta _{\mathrm{B}}^2+\Delta _{\mathrm{C}}^2)}{U}$.

The superfluid density can be obtained with phase twist method. Assuming the superfluid order parameters undergo a phase variation, e.g, $\triangle (\mathbf{r })\rightarrow \Delta (\mathbf{r })e^{2i\mathbf{q }\cdot \mathbf{r }}$, the superfluid density tensor $\rho _{sij}$ can be written as

45 $$\begin{aligned} \rho _{sij}= \frac{\partial ^2 \Omega (\mathbf{q })}{\partial q_i \partial q_i}|_{q\rightarrow 0}, \end{aligned}$$

where $\Omega (\mathbf{q })$ is thermodynamical potential. In the presence of phase twist, the BdG Hamiltonian (after applying a gauge transformation) becomes

46 $$\begin{aligned} H_{\mathrm{BdG}}= \sum _\mathbf{{k}} \Psi ^{\dag }(\mathbf{k }) H_{\mathrm{BdG}}(\mathbf{k,q})\Psi (\mathbf{k }), \end{aligned}$$

where $\Psi ^\dagger (\mathbf{k})\!\!=\!\![\psi _{A\uparrow }^\dagger (\mathbf{k}),\psi _{B\uparrow }^\dagger (\mathbf{k}),\psi _{C\uparrow }^\dagger (\mathbf{k}),\psi _{A\downarrow }(-\mathbf{k}),\psi _{B\downarrow }(-\mathbf{k}),\psi _{C\downarrow }(-\mathbf{k})]$ and the BdG matrix

47 $$\begin{aligned}&H_{\mathrm{BdG}}(\mathbf{k,q})=\left( \!\!\! \begin{array}{cccccc} h^{p}_{11}(\mathbf{k }+\mathbf{q })&{} h^{p}_{12}(\mathbf{k }+\mathbf{q }) &{} 0&{} \Delta _A &{}0 &{} 0 \\ h^{p}_{21}(\mathbf{k }+\mathbf{q }) &{} h^{p}_{22}(\mathbf{k }+\mathbf{q }) &{}h^{p}_{23}(\mathbf{k }+\mathbf{q }) &{} 0&{}\Delta _B &{} 0 \\ 0 &{} h^{p}_{23}(\mathbf{k }+\mathbf{q }) &{}h^{p}_{33}(\mathbf{k }+\mathbf{q }) &{} 0 &{} 0 &{}\Delta _C\\ \Delta ^{*}_{A} &{}0 &{} 0 &{}-h^{h}_{11}(-\mathbf{k }+\mathbf{q }) &{} -h^{h}_{12}(-\mathbf{k }+\mathbf{q })&{}0 \\ 0 &{}\Delta ^{*}_{B} &{} 0 &{}-h^{h}_{21}(-\mathbf{k }+\mathbf{q }) &{} -h^{h}_{22}(-\mathbf{k }+\mathbf{q })&{}-h^{h}_{23}(-\mathbf{k }+\mathbf{q }) \\ 0 &{}0 &{} \Delta ^{*}_{C} &{}0 &{} -h^{h}_{32}(-\mathbf{k }+\mathbf{q })&{}-h^{h}_{33}(-\mathbf{k }+\mathbf{q }) \\ \end{array}\!\!\!\right) . \end{aligned}$$

After diagonalizing the above BdG matrix, we can get the eigen-energies $E^{n+}(\mathbf {k,q})$ and $E^{n-}(\mathbf {k,q})$. The thermodynamical potential becomes

48 $$\begin{aligned} \Omega (\mathbf{q })=\frac{1}{N_{cell}}\sum _{\mathbf{k },n=1,2,3} E^{n-}(\mathbf{k },\mathbf{q })+Cons. \end{aligned}$$

In addition, if a Hamiltonian *H*, its eigen-states $|n\rangle$ and eigen-energies $E^n$ depend on parameters $\mathbf{k }$ and $\mathbf{q }$, they should satisfy

49 $$\begin{aligned}&H|n\rangle =E^{n}|n\rangle ,\nonumber \\&\langle m|n\rangle =\delta _{mn},\nonumber \\&E^n=\langle n |H|n\rangle ,\nonumber \\&\frac{\partial E^n}{\partial q_i}=\langle n|\frac{\partial H}{\partial q_i}|n\rangle ,\nonumber \\&\frac{\partial E^n}{\partial k_i}=\langle n|\frac{\partial H}{\partial k_i}|n\rangle ,\nonumber \\&\langle m|\frac{\partial n}{\partial q_i}\rangle =\frac{\langle m|\frac{\partial H}{\partial q_i}|n\rangle }{E^n-E^m},\nonumber \\&\langle m|\frac{\partial n}{\partial k_i}\rangle =\frac{\langle m|\frac{\partial H}{\partial k_i}|n\rangle }{E^n-E^m},\nonumber \\&\frac{\partial ^2 E^n}{\partial q_i \partial q_j}=\langle n|\frac{\partial ^2 H}{\partial q_i \partial q_j}|n\rangle +\sum _{m\ne n}\left[ \frac{\langle n|\frac{\partial H}{\partial q_i}|m\rangle \langle m|\frac{\partial H}{\partial q_j}|n\rangle }{E^n-E^m}+\frac{\langle n|\frac{\partial H}{\partial q_j}|m\rangle \langle m|\frac{\partial H}{\partial q_i}|n\rangle }{E^n-E^m}\right] ,\nonumber \\&\frac{\partial ^2 E^n}{\partial k_i \partial k_j}=\langle n|\frac{\partial ^2 H}{\partial k_i \partial k_j}|n\rangle +\sum _{m\ne n}\left[ \frac{\langle n|\frac{\partial H}{\partial k_i}|m\rangle \langle m|\frac{\partial H}{\partial k_j}|n\rangle }{E^n-E^m}+\frac{\langle n|\frac{\partial H}{\partial k_j}|m\rangle \langle m|\frac{\partial H}{\partial k_i}|n\rangle }{E^n-E^m}\right] . \end{aligned}$$

Due to $\langle n|\frac{\partial ^2 H_{\mathrm{BdG}}(\mathbf{k,q})}{\partial k_i \partial k_j}|n\rangle =\langle n|\frac{\partial ^2 H_{\mathrm{BdG}}(\mathbf{k,q})}{\partial q_i \partial q_j}|n\rangle$ for the BdG matrix $H_{\mathrm{BdG}}(\mathbf{k,q})$, so the superfluid density can be written as

50 $$\begin{aligned} \rho _{sij}&=\frac{1}{N_{cell}}\sum _{\mathbf{k }nm}\frac{\partial ^2 E^{n-}}{\partial k_i \partial k_j}|_{q\rightarrow 0}\nonumber \\&\quad +\frac{1}{N_{cell}}\sum _{\mathbf{k }nm}\left\{ \left[ \frac{\langle n-|\frac{\partial H}{\partial q_i}|m+\rangle \langle m+|\frac{\partial H}{\partial q_j}|n-\rangle }{E^{n-}-E^{m+}}+\frac{\langle n-|\frac{\partial H}{\partial q_j}|m+\rangle \langle m+|\frac{\partial H}{\partial q_i}|n-\rangle }{E^{n-}-E^{m+}}\right] \right. \nonumber \\&\quad -\left. \left[ \frac{\langle n-|\frac{\partial H}{\partial k_i}|m+\rangle \langle m+|\frac{\partial H}{\partial k_j}|n-\rangle }{E^{n-}-E^{m+}}+\frac{\langle n-|\frac{\partial H}{\partial k_j}|m+\rangle \langle m+|\frac{\partial H}{\partial k_i}|n-\rangle }{E^{n-}-E^{m+}}\right] \right\} _{q\rightarrow 0}, \end{aligned}$$

where we use *H* to denote the BdG matrix $H_{\mathrm{{BdG}}}(\mathbf {k,q})$. $|m+\rangle \equiv |m+,\mathbf{k },\mathbf{q }\rangle$ ($|n-\rangle \equiv |n-,\mathbf{k },\mathbf{q }\rangle$) and $E^{m+}\equiv E^{m+}(\mathbf{k },\mathbf{q })[E^{n-}\equiv E^{n-}(\mathbf{k },\mathbf{q })]$ are eigen-states and eigen-energies of Hamiltonian $H_{\mathrm{{BdG}}}(\mathbf {k,q})$. The first term of superfluid density depends on the second derivative $\frac{\partial ^2 E^{n-}}{\partial k_i \partial k_j}$, it would vanish. Because quasi-particle energy $E^{n-}(k_x,k_y)$ is a periodic function of $k_x$, the summation over *k* can be transformed into an integral. An integral of a derivative of periodic function over a period is zero. So the superfluid density is

51 $$\begin{aligned}&\rho _{sij}= \frac{\partial ^2 \Omega (\mathbf{q })}{\partial q_i \partial q_i}=\frac{1}{N_{cell}}\sum _{\mathbf{k }nm}\left[ \frac{\langle n-|\frac{\partial H}{\partial q_i}|m+\rangle \langle m+|\frac{\partial H}{\partial q_j}|n-\rangle }{E^{n-}-E^{m+}}-\frac{\langle n-|\frac{\partial H}{\partial k_i}|m+\rangle \langle m+|\frac{\partial H}{\partial k_j}|n-\rangle }{E^{n-}-E^{m+}}+(i\leftrightarrow j)\right] _{q\rightarrow 0}. \end{aligned}$$

When $$q\rightarrow 0$$,

52 $$\begin{aligned} \frac{\partial H}{\partial q_i}=\left( \!\!\! \begin{array}{cccc} [\frac{\partial h^{p}(\mathbf{k })}{\partial k_i}]_{3\times 3} &{}(0)_{3\times 3} \\ (0)_{3\times 3} &{} [\frac{\partial h^{h}(-\mathbf{k })}{\partial k_i}]_{3\times 3} \\ \end{array}\!\!\!\right) , \end{aligned}$$

and

53 $$\begin{aligned} \frac{\partial H}{\partial k_i}=\left( \!\!\! \begin{array}{cccc} [\frac{\partial h^{p}(\mathbf{k })}{\partial k_i}]_{3\times 3} &{}(0)_{3\times 3} \\ (0)_{3\times 3} &{} -[\frac{\partial h^{h}(-\mathbf{k })}{\partial k_i}]_{3\times 3} \\ \end{array}\!\!\!\right) . \end{aligned}$$

Using the energy-band basis, the superfluid density

54 $$\begin{aligned}&\rho _{sij}=\frac{2}{N_{cell}}\sum _{\mathbf{k }nm s_1s_2t_1t_2}\left[ \frac{W^{-*}_{ns_1}W^{+}_{ms_2}W^{+*}_{mt_1}W^{-}_{nt_2}}{E^{n-}-E^{m+}} \left\langle s_1p|\frac{\partial h^{p}}{\partial k_i}|s_2 p \right\rangle \left\langle t_1 h|\frac{\partial h^{h}}{\partial k_j}|t_2 h \right\rangle \right. \nonumber \\&\quad \left. +\frac{W^{-*}_{nt_1}W^{+}_{mt_2}W^{+*}_{ms_1}W^{-}_{ns_2}}{E^{n-}-E^{m+}} \left\langle t_1 h|\frac{\partial h^{h}}{\partial k_i}|t_2h \right\rangle \left\langle s_1p|\frac{\partial h^{p}}{\partial k_j}|s_2 p \right\rangle +(i\leftrightarrow j)\right] . \end{aligned}$$

When interaction $U\rightarrow \infty$, the chemical potential $\mu \propto U$ and the order parameters $\Delta _A\simeq \Delta _B\simeq \Delta _C\propto U$, which corresponds to the uniform pairing case^20^. In such a case, the superfluidity takes place independently in the respective energy bands (no inter-band pairings). The transformation matrix takes the following form

55 $$\begin{aligned}W^{+}_{ns}(\mathbf{k })\simeq \delta _{ns}\sqrt{\frac{1}{2}\left( 1+\frac{\varepsilon _{n}(\mathbf{k })}{E^{n}(\mathbf{k })}\right) },\nonumber \\W^{+}_{nt}(\mathbf{k })\simeq \delta _{nt}\sqrt{\frac{1}{2}\left( 1-\frac{\varepsilon _{n}(\mathbf{k })}{E^{n}(\mathbf{k })}\right) }. \end{aligned}$$

In addition, the particle-hole symmetry of BdG matrix implies that the transformation matrices satisfy

56 $$\begin{aligned}W^{-}_{ns}(\mathbf{k })=\delta _{st}\delta _{ns}W^{+*}_{nt}(\mathbf{k })=\delta _{st}\delta _{ns}\sqrt{\frac{1}{2}\left( 1-\frac{\varepsilon _{n}(\mathbf{k })}{E^{n}(\mathbf{k })}\right) },\nonumber \\W^{-}_{nt}(\mathbf{k })=-\delta _{st}\delta _{ns}W^{+*}_{ns}(\mathbf{k })=-\delta _{st}\delta _{ns}\sqrt{\frac{1}{2}\left( 1+\frac{\varepsilon _{n}(\mathbf{k })}{E^{n}(\mathbf{k })}\right) }. \end{aligned}$$

When $U\rightarrow \infty$, the order parameter and chemical potential

57 $$\begin{aligned}\Delta \simeq \frac{U}{6}\sqrt{n(6-n)},\nonumber \\\mu \simeq \frac{U}{6}(n-3), \end{aligned}$$

where *n* is filling factor (particle number per unit cell). In addition, the quasi-particle energies satisfy

58 $$\begin{aligned} E^{n+}(\mathbf{k })=-E^{n-}(-\mathbf{k })=\sqrt{(\varepsilon _{n}(\mathbf{k }))^2+\Delta ^2}\simeq \sqrt{\mu ^2+\Delta ^2};\quad E^{n-}-E^{m+}\simeq -2\sqrt{\mu ^2+\Delta ^2}, \end{aligned}$$

where $\varepsilon _{n}(\mathbf{k })\simeq -\mu$ as $U\rightarrow \infty$. The superfluid density is reduced to

59 $$\begin{aligned}&\rho _{sij}\simeq \frac{\Delta ^2}{2N_{cell}(\mu ^2+\Delta ^2)^{3/2}}\sum _{\mathbf{k }nm}\left[ \langle n|\frac{\partial h(\mathbf {k})}{\partial k_i}|m\rangle \langle m|\frac{\partial h(\mathbf {k})}{\partial k_j}|n\rangle +\langle n|\frac{\partial h(\mathbf {k})}{\partial k_j}|m\rangle \langle m|\frac{\partial h(\mathbf {k})}{\partial k_i}|n\rangle \right] , \end{aligned}$$

where $|n\rangle \equiv |n,\mathbf{k }\rangle$ is energy band basis, and $h(\mathbf {k})$ is single-particle Hamiltonian (Eq. 3).

So the superfluid density

60 $$\begin{aligned}&\rho _{sij}\simeq \frac{\Delta ^2}{2N_{cell}(\mu ^2+\Delta ^2)^{3/2}}\sum _{\mathbf{k }nm}\left[ \langle n|\frac{\partial h(\mathbf {k})}{\partial k_i}|m\rangle \langle m|\frac{\partial h(\mathbf {k})}{\partial k_j}|n\rangle +\langle n|\frac{\partial h(\mathbf {k})}{\partial k_j}|m\rangle \langle m|\frac{\partial h(\mathbf {k})}{\partial k_i}|n\rangle \right] ,\nonumber \\&=\frac{2n(6-n)}{9U}S_{ij}, \end{aligned}$$

where

61 $$\begin{aligned}&S_{ij}=\frac{1}{2N_{cell}}\sum _{\mathbf{k }nm}\left[ \langle n|\frac{\partial h(\mathbf {k})}{\partial k_i}|m\rangle \langle m|\frac{\partial h(\mathbf {k})}{\partial k_j}|n\rangle +\langle n|\frac{\partial h(\mathbf {k})}{\partial k_j}|m\rangle \langle m|\frac{\partial h(\mathbf {k})}{\partial k_i}|n\rangle \right] , \end{aligned}$$

is a rank-two tensor, which only depends on single-particle Hamiltonian. For dice lattices in our work, $S_{ij}=6t^2\delta _{ij}$. So the asymptotic formula for superfluid density (particle number per unit cell) is obtained ($U\rightarrow \infty$)

62 $$\begin{aligned} \rho _{sij}\simeq \frac{4t^2n(6-n)}{3U}\delta _{ij}. \end{aligned}$$

### Gaussian fluctuation method

To investigate the collective modes, it is very convenient to formulate the theory with functional integration. The partition function of grand canonical ensemble is written as functional integral of Grassman fields ${\bar{\psi }}$ and $\psi$

63 $$\begin{aligned} Z \equiv \int D[{\bar{\psi }}_{iA\sigma }{\bar{\psi }}_{iB\sigma }{\bar{\psi }}_{iC\sigma }]D[\psi _{iA\sigma }\psi _{iB\sigma }\psi _{iC\sigma }] e^{-S}, \end{aligned}$$

where we have the action *S* given by

$$\begin{aligned} S=\sum _{iA,\sigma ;iB,\sigma ;iC,\sigma }\int _{0}^{\beta }d\tau \left[ {\bar{\psi }}_{iA\sigma }(\tau )\frac{\partial \psi _{iA\sigma }(\tau )}{\partial \tau }+{\bar{\psi }}_{iB\sigma }(\tau )\frac{\partial \psi _{iB\sigma }(\tau )}{\partial \tau }+{\bar{\psi }}_{iC\sigma }(\tau )\frac{\partial \psi _{iC\sigma }(\tau )}{\partial \tau } +H(\psi _{iA\sigma },\psi _{iB\sigma },\psi _{iC\sigma })\right] , \end{aligned}$$

where the inverse temperature $\beta =1/T$. In this paper, we will only focus on the properties at zero temperature and will take $\beta \rightarrow \infty$ at the end. By introducing the Hubbard-Stratanovich fields $\Delta _{iA(B,C)}$ for sub-lattice **A(B,C)** as usual, we have

64 $$\begin{aligned} Z =\int D\Delta ^{*}D \Delta D {\bar{\psi }} D \psi e^{-S_\Delta }, \end{aligned}$$

and the effective action is given by

65 $$\begin{aligned} S_{\Delta } = \int _{0}^{\beta }d\tau \sum _{Ai,\sigma ;Bj,\sigma }\left[ \frac{|\Delta _{iA\sigma }(\tau )|^2}{U}+\frac{|\Delta _{iB\sigma }(\tau )|^2}{U}+\frac{|\Delta _{iC\sigma }(\tau )|^2}{U}- {\bar{\psi }} G^{-1}(\Delta _{iA}(\tau ),\Delta _{iB}(\tau ),\Delta _{iC}(\tau ))\psi \right] . \end{aligned}$$

In the following, we will use the subscribe $i_1$, and $i'_{1}$ [($i_{2}$, $i'_{2}$)/($i_{3}$, $i'_{3}$)] to denote the positions of sub-lattices **A** [**B**/**C**] unless stated otherwise. With the basis ${\bar{\psi }}=[{\bar{\psi }}_{i_1A\uparrow },{\bar{\psi }}_{i_2B\uparrow },{\bar{\psi }}_{i_3C\uparrow };\psi _{i_1A\downarrow },\psi _{i_2B\downarrow },\psi _{i_3C\downarrow }]$, the inverse Nambu–Gorkov Green function $G^{-1}$ takes following form

66 $$\begin{aligned}&G^{-1}=\left( \begin{array}{cccccc} (A)_{i_1,i'_1} &{} (A)_{i_1,i_2} &{}0 &{} (B)_{i_1,i'_1} &{}0 &{} 0\\ (A)^{*}_{i_1,i_2} &{} (A)_{i_2,i'_2} &{} (A)_{i_2,i_3} &{} 0 &{} (B)_{i_2,i'_2}&{}0\\ 0 &{} (A)^{*}_{i_2,i_3} &{} (A)_{i_3,i'_3} &{} 0 &{} 0&{}(B)_{i_3,i'_3}\\ (B)^*_{i_1,i'_1} &{} 0 &{}0 &{} (D)_{i_1,i'_1} &{} (D)_{i_1,i_2}&{} 0\\ 0 &{} (B)^*_{i_2,i'_2} &{}0 &{} (D)^{*}_{i_1,i_2} &{} (D)_{i_2,i'_2} &{}(D)_{i_2,i_3} \\ 0 &{} 0&{} (B)^*_{i_3,i'_3} &{} 0 &{} (D)^{*}_{i_2,i_3} &{}(D)_{i_3,i'_3} \\ \end{array}\right) , \end{aligned}$$

where

67 $$\begin{aligned} A_{i_1,i'_1}&=(-\partial _\tau +\mu )\delta _{i_1,i'_1}\delta (\tau -\tau '),\nonumber \\ A_{i_1,i_2}&= -t\sum _\delta \delta _{i_1,i_2-\mathbf{a }_\delta }\delta (\tau -\tau '),\nonumber \\ A_{i_2,i'_2}&= (-\partial _\tau +\mu )\delta _{i_2,i'_2}\delta (\tau -\tau '),\nonumber \\ A_{i_2,i_3}&= -t\sum _\delta \delta _{i_2,i_3-\mathbf{a }_\delta }\delta (\tau -\tau '),\nonumber \\ A_{i_3,i'_3}&= (-\partial _\tau +\mu )\delta _{i_3,i'_3}\delta (\tau -\tau '),\nonumber \\ B_{i_1,i'_1}&= \Delta _{i_1A}(\tau )\delta _{i_1,i'_1}\delta (\tau -\tau '),\nonumber \\ B_{i_2,i'_2}&= \Delta _{i_2B}(\tau )\delta _{i_2,i'_2}\delta (\tau -\tau '),\nonumber \\ B_{i_3,i'_3}&= \Delta _{i_3C}(\tau )\delta _{i_3,i'_3}\delta (\tau -\tau '),\nonumber \\ D_{i_1,i'_1}&= (-\partial _\tau -\mu )\delta _{i_1,i'_1}\delta (\tau -\tau '),\nonumber \\ D_{i_1,i_2}&= t\sum _\delta \delta _{i_1,i_2-\mathbf{a }_\delta }\delta (\tau -\tau '),\nonumber \\ D_{i_2,i'_2}&= (-\partial _\tau -\mu )\delta _{i_2,i'_2}\delta (\tau -\tau '),\nonumber \\ D_{i_2,i_3}&= t\sum _\delta \delta _{i_2,i_3-\mathbf{a }_\delta }\delta (\tau -\tau '),\nonumber \\ D_{i_3,i'_3}&= (-\partial _\tau -\mu )\delta _{i_3,i'_3}\delta (\tau -\tau '). \end{aligned}$$

The functional integral is quadratic with respect to $\psi$ fields, we can integrate it out

68 $$\begin{aligned} Z&= \int D\Delta ^{*}D \Delta \exp \left\{-\int ^{\beta }_{0} d\tau \sum _{i} \left[\frac{|\Delta _{iA}(\tau )|^2}{U}+\frac{|\Delta _{iB}(\tau )|^2}{U}+\frac{|\Delta _{iC}(\tau )|^2}{U}-\mathrm{Tr}\ln G^{-1}(\Delta _{iA}(\tau ),\Delta _{iB}(\tau ),,\Delta _{iC}(\tau ))\right] \right\}. \end{aligned}$$

Further assuming $\Delta _{iA}$, $\Delta _{iB}$ and $\Delta _{iC}$ can be written as $\Delta _{iA}(\tau )=\Delta _A+\delta \Delta _{iA}(\tau )$, $\Delta _{iB}(\tau )=\Delta _B+\delta \Delta _{iB}(\tau )$ and $\Delta _{iC}(\tau )=\Delta _C+\delta \Delta _{iC}(\tau )$, where $\Delta _{A(B/C)}$ are not dependent on spatial and time variables. It is convenient to write the $G^{-1}$ in momentum and Matsubara frequency spaces, i.e.,

69 $$\begin{aligned}G^{-1}(\mathbf{k },\mathbf{k }')=G^{-1}_{0}(\mathbf{k },\mathbf{k }')+K(\mathbf{k },\mathbf{k }'), \end{aligned}$$

where

70 $$\begin{aligned}G_{0}^{-1}(\mathbf{k },\mathbf{k }')=\left( \begin{array}{cccccc} A_{1,1} &{} A_{1,2} &{}0 &{} \Delta _A &{} 0&{} 0\\ A^{*}_{1,2} &{} A_{2,2} &{} A_{2,3} &{} 0 &{} \Delta _B &{} 0 \\ 0 &{} A^{*}_{2,3} &{} A_{2,3} &{} 0 &{} 0 &{} \Delta _C \\ \Delta ^{*}_{A} &{} 0&{}0 &{} D_{1,1} &{} D_{1,2}&{}0\\ 0 &{} \Delta ^{*}_{B} &{}0 &{} D^{*}_{1,2} &{} D_{2,2}&{}D_{2,3} \\ 0 &{} 0 &{}\Delta ^{*}_{C} &{} 0 &{} D^{*}_{2,3}&{}D_{3,3} \\ \end{array}\right) \delta (\mathbf{k }-\mathbf{k }'), \end{aligned}$$

and

71 $$\begin{aligned}K (\mathbf{k },\mathbf{k }')=\left( \!\!\! \begin{array}{cccccc} 0 &{} 0 &{} 0&{} \Delta _A(-k'+k) &{} 0 &{}0\\ 0 &{} 0 &{} 0&{} 0 &{} \Delta _B(-k'+k)&{}0 \\ 0 &{} 0 &{} 0&{} 0 &{} 0&{}\Delta _C(-k'+k) \\ \Delta _{A}^{*}(k'-k) &{}0&{}0 &{} 0 &{} 0 &{} 0\\ 0 &{} \Delta _{B}^{*}(k'-k) &{}0 &{} 0 &{} 0&{}0 \\ 0 &{} 0 &{}\Delta _{C}^{*}(k'-k) &{} 0 &{} 0&{}0 \\ \end{array} \!\!\!\right) , \end{aligned}$$

where we introduce $\delta (\mathbf{k }-\mathbf{k }')\equiv \delta ^{2}(\mathbf{k }-\mathbf{k }')\delta _{n,n'}$, $k=(\mathbf{k },i\omega _n)$, $k'=(\mathbf{k }',i\omega _{n'})$, $\delta k=k'-k=(\mathbf{k }'-\mathbf{k },i\omega _{n'}-i\omega _n)$ and

72 $$\begin{aligned}&A_{1,1}=i\omega _n+\mu ,\nonumber \\&A_{1,2}=-t\sum _\delta e^{i\mathbf{k }\cdot \mathbf{a }_\delta },\nonumber \\&A_{2,2}=i\omega _n+\mu ,\nonumber \\&A_{2,3}=-t\sum _\delta e^{i\mathbf{k }\cdot \mathbf{a }_\delta },\nonumber \\&A_{3,3}=i\omega _n+\mu ,\nonumber \\&D_{1,1}=i\omega _n-\mu ,\nonumber \\&D_{1,2}=t\sum _\delta e^{i\mathbf{k }\cdot \mathbf{a }_\delta },\nonumber \\&D_{2,2}=i\omega _n-\mu ,\nonumber \\&D_{2,3}=t\sum _\delta e^{i\mathbf{k }\cdot \mathbf{a }_\delta },\nonumber \\&D_{3,3}=i\omega _n-\mu . \end{aligned}$$

Expanding action $S_\Delta$ to second order of $\delta \Delta$ , one gets the thermodynamic potential

73 $$\begin{aligned} Z\approx e^{-S_0}\int D {\bar{\eta }}_q D\eta _q e^{-\delta S}, \end{aligned}$$

where $S_0$ is the action from the mean-field contribution and $q=\delta k$. The linear terms vanish due to the mean-field (saddle-point) equations. The fluctuation contribution is

74 $$\begin{aligned} \delta S=\frac{1}{2}\sum _{\mathbf{q },n}\bar{\eta _q}M\eta _q=\sum _{\mathbf{q },n>0}\bar{\eta _q}M\eta _q, \end{aligned}$$

where

$$\begin{aligned} {\bar{\eta }}_\mathbf{q}\mathbf =[\Delta ^{*}_{A}(\mathbf{q },i\omega _n),\Delta ^{*}_{B}(\mathbf{q },i\omega _n),\Delta ^{*}_{C}(\mathbf{q },i\omega _n);\Delta _{A}(-\mathbf{q },-i\omega _n),\Delta _{B}(-\mathbf{q },-i\omega _n),\Delta _{C}(-\mathbf{q },-i\omega _n)] \end{aligned}$$

and the fluctuation matrix *M* is given by

75 $$  \begin{aligned}&M_{ij}(\mathbf{q },i\omega _n)=\frac{1}{\beta }\sum _{\mathbf{k },n'}G^{0}_{ij}(k+q)G^{0}_{j+m,i+m}(k)+\frac{\delta _{ij}}{U}, \qquad \qquad \qquad \qquad (1\le i,j\le m)\nonumber \\&M_{ij}(\mathbf{q },i\omega _n)=\frac{1}{\beta }\sum _{\mathbf{k },n'}G^{0}_{ij}(k+q)^{0}G_{j-m,i+m}(k), \qquad \qquad \qquad \qquad (1\le i\le m \; \& \; m+1\le j\le 2m)\nonumber \\&M_{ij}(\mathbf{q },i\omega _n)=\frac{1}{\beta }\sum _{\mathbf{k },n'}G^{0}_{ij}(k+q)G^{0}_{j+m,i-m}(k), \qquad \qquad \qquad \qquad (m+1\le i\le 2m \;  \&  \; 1\le j\le m)\nonumber \\&M_{ij}(\mathbf{q },i\omega _n)=\frac{1}{\beta }\sum _{\mathbf{k },n'}G^{0}_{ij}(k+q)G^{0}_{j-m,i-m}(k)+\frac{\delta _{ij}}{U}, \qquad \qquad \qquad \qquad (m+1\le i,j\le 2m ), \end{aligned}$$

where the number of order parameters $m=3$ and Green function $G^{0}_{ij}(\mathbf{k },i\omega _{n'})= ([i\omega _{n'}-H_{\mathrm{BdG}}(\mathbf{k })]^{-1})_{ij}$ is matrix element of Nambu–Gorkov Green function. The collective modes are given by zeroes of determinant $\mathrm{Det}|M(\mathbf{q },i\omega _n\rightarrow \omega +i0^+)|=0$.

## References

[1] Peotta S, Törmä P (2015). Superfluidity in topologically nontrivial flat bands. Nat. Commun..

[2] Iskin M (2020). Collective excitations of a BCS superfluid in the presence of two sublattices. Phys. Rev. A.

[3] Cao Y (2018). Unconventional superconductivity in magic-angle graphene superlattices. Nature.

[4] Wu YR, Zhang YC (2021). Superfluid states in $$\alpha -t_3$$ lattice. Chin. Phys. B.

[5] Leggett AJ (1966). Number-phase fluctuations in two-band superconductors. Prog. Theor. Phys..

[6] Iskin M, Sáde Melo CAR (2005). Bcs-bec crossover of collective excitations in two-band superfluids. Phys. Rev. B.

[7] He L, Wang J, Peng S-G, Liu X-J, Hu H (2016). Strongly correlated Fermi superfluid near an orbital Feshbach resonance: Stability, equation of state, and Leggett mode. Phys. Rev. A.

[8] Zhang Y-C, Ding S, Zhang S (2017). Collective modes in a two-band superfluid of ultracold alkaline-earth-metal atoms close to an orbital Feshbach resonance. Phys. Rev. A.

[9] Klimin SN, Kurkjian H, Tempere J (2019). Leggett collective excitations in a two-band fermi superfluid at finite temperatures. New J. Phys..

[10] Honerkamp C, Hofstetter W (2004). Ultracold fermions and the SU(N) hubbard model. Phys. Rev. Lett..

[11] Rapp A, Zaránd G, Honerkamp C, Hofstetter W (2007). Color superfluidity and baryon formation in ultracold fermions. Phys. Rev. Lett..

[12] Liu X-J, Hu H, Drummond PD (2008). Multicomponent strongly attractive fermi gas: A color superconductor in a one-dimensional harmonic trap. Phys. Rev. A.

[13] Cherng RW, Refael G, Demler E (2007). Superfluidity and magnetism in multicomponent ultracold fermions. Phys. Rev. Lett..

[14] Kanász-Nagy M, Zaránd G (2012). Global superfluid phase diagram of a three-component fermion mixture with magnetic ordering. Phys. Rev. B.

[15] Martikainen J-P, Kinnunen JJ, Törmä P, Pethick CJ (2009). Induced interactions and the superfluid transition temperature in a three-component fermi gas. Phys. Rev. Lett..

[16] Zhu Q, Zhang C, Wu B (2012). Exotic superfluidity in spin-orbit coupled Bose–Einstein condensates. Europhys. Lett..

[17] Hazra T, Verma N, Randeria M (2019). Bounds on the superconducting transition temperature: Applications to twisted bilayer graphene and cold atoms. Phys. Rev. X.

[18] Julku A, Peotta S, Vanhala TI, Kim D-H, Törmä P (2016). Geometric origin of superfluidity in the lieb-lattice flat band. Phys. Rev. Lett..

[19] Julku A, Peltonen TJ, Liang L, Heikkilä TT, Törmä P (2020). Superfluid weight and Berezinskii–Kosterlitz–Thouless transition temperature of twisted bilayer graphene. Phys. Rev. B.

[20] Liang L (2017). Band geometry, berry curvature, and superfluid weight. Phys. Rev. B.

[21] Hu X, Hyart T, Pikulin DI, Rossi E (2019). Geometric and conventional contribution to the superfluid weight in twisted bilayer graphene. Phys. Rev. Lett..

[22] Xie F, Song Z, Lian B, Bernevig BA (2020). Topology-bounded superfluid weight in twisted bilayer graphene. Phys. Rev. Lett..

[23] Provost JP, Vallee G (1980). Riemannian structure on manifolds of quantum states. Commun. Math. Phys..

[24] Sutherland B (1986). Localization of electronic wave functions due to local topology. Phys. Rev. B.

[25] Vidal J, Mosseri R, Doucot B (1998). Aharonov–Bohm cages in two-dimensional structures. Phys. Rev. Lett..

[26] Gorbar EV, Gusynin VP, Oriekhov DO (2019). Electron states for gapped pseudospin-1 fermions in the field of a charged impurity. Phys. Rev. B.

[27] Dora B, Kailasvuori J, Moessner R (2011). Lattice generalization of the dirac equation to general spin and the role of the flat band. Phys. Rev. B.

[28] Xu H-Y, Lai Y-C (2020). Anomalous chiral edge states in spin-1 dirac quantum dots. Phys. Rev. Res..

[29] Wang F, Ran Y (2011). Nearly flat band with Chern number c=2 on the dice lattice. Phys. Rev. B.

[30] Bercioux D, Urban DF, Grabert H, Häusler W (2009). Massless Dirac–Weyl fermions in a $$t_3$$ optical lattice. Phys. Rev. A.

[31] Illes E, Nicol EJ (2016). Magnetic properties of the $$\alpha -t_3$$ model: Magneto-optical conductivity and the hofstadter butterfly. Phys. Rev. B.

[32] Chen Y-R, Xu Y, Wang J, Liu J-F, Ma Z (2019). Enhanced magneto-optical response due to the flat band in nanoribbons made from the $$\alpha -t_3$$ lattice. Phys. Rev. B.

[33] Illes E, Nicol EJ (2017). Klein tunneling in the $$\alpha -t_3$$ model. Phys. Rev. B.

[34] Biswas T, Ghosh TK (2018). Dynamics of a quasiparticle in the $$\alpha -t_3$$ model: Role of pseudospin polarization and transverse magnetic field on zitterbewegung. J. Phys. Condens. Matter.

[35] Raoux A, Morigi M, Fuchs J-N, Piéchon F, Montambaux G (2014). From dia- to paramagnetic orbital susceptibility of massless fermions. Phys. Rev. Lett..

[36] Biswas T, Ghosh T (2016). Magnetotransport properties of the $$\alpha -t_3$$ model. J. Phys. Condensed Matter.

[37] Islam SF, Dutta P (2017). Valley-polarized magnetoconductivity and particle-hole symmetry breaking in a periodically modulated $$\alpha -t_3$$ lattice. Phys. Rev. B.

[38] Blumberg G (2007). Observation of Leggett’s collective mode in a multi-band mgb$$_2$$ superconductor. Phys. Rev. Lett..

[39] Mou D (2015). Strong interaction between electrons and collective excitations in the multiband superconductor mgb$$_2$$. Phys. Rev. B.

[40] Zhang Y-C, Xu Z, Zhang S (2017). Topological superfluids and the bec-bcs crossover in the attractive Haldane–Hubbard model. Phys. Rev. A.

[41] Heiselberg H (2017). Crossovers in Unitary Fermi Systems, bcs-bec Crossover and the Unitary Fermi Gas, edited by W. Zwerger.

[42] Iglovikov VI, Hébert F, Grémaud B, Batrouni GG, Scalettar RT (2014). Superconducting transitions in flat-band systems. Phys. Rev. B.

[43] Wu C, Sarma SD (2008). $$p_{x, y}$$-orbital counterpart of graphene: Cold atoms in the honeycomb optical lattice. Phys. Rev. B.

[44] Zhang S, Hung H-H, Wu C (2010). Proposed realization of itinerant ferromagnetism in optical lattices. Phys. Rev. A.

[45] Kopnin NB, Heikkila TT, Volovik GE (2011). High-temperature surface superconductivity in topological flat-band systems. Phys. Rev. B.

[46] Fisher ME, Barber MN, Jasnow D (1973). Helicity modulus, superfluidity, and scaling in isotropic systems. Phys. Rev. A.

[47] Zhang, Y.-C. Superfluid density, josephson relation and pairing fluctuations in a multi-component fermion superfluid. arXiv:2012.11217 (2020). 10.1038/s41598-021-01261-y PMC8575947 34750432

[48] Pieri P, Strinati GC (2000). Strong-coupling limit in the evolution from bcs superconductivity to Bose–Einstein condensation. Phys. Rev. B.

[49] Zhang YC (2016). Superfluid density of a spin-orbit-coupled Bose gas. Phys. Rev. A.

[50] Zhang YC, Wang HT, Shen SQ, Liu WM (2013). Particle-hole bound states of dipolar molecules in optical lattice. Chin. Phys. B.

[51] Iskin M (2019). Origin of flat-band superfluidity on the Mielke checkerboard lattice. Phys. Rev. A.

[52] Onogi T, Murayama Y (1994). Two-dimensional superfluidity and localization in the hard core boson model: A quantum Monte Carlo study. Phys. Rev. B.

[53] Micnas R, Robaszkiewicz S, Kostyrko T (1995). Thermodynamic and electromagnetic properties of hard-core charged bosons on a lattice. Phys. Rev. B.

[54] Engelbrecht JR, Randeria M, Sáde Melo CAR (1997). Bcs to Bose crossover: Broken-symmetry state. Phys. Rev. B.

[55] Hu H, Liu X-J, Drummond PD (2006). Equation of state of a superfluid fermi gas in the bcs-bec crossover. Europhys. Lett..

[56] Diener RB, Sensarma R, Randeria M (2008). Quantum fluctuations in the superfluid state of the bcs-bec crossover. Phys. Rev. A.

[57] Samanta A, Ratnakar A, Trivedi N, Sensarma R (2020). Two-particle spectral function for disordered s-wave superconductors: Local maps and collective modes. Phys. Rev. B.

[58] White SR (1991). Spectral weight function for the two-dimensional Hubbard model. Phys. Rev. B.

[59] Zhang Y-C (2018). Generalized Josephson relation for conserved charges in multicomponent bosons. Phys. Rev. A.

[60] Lifshitz EM, Pitaevskii LP (1980). Statistical Physics Part 2.

